# Midbrain dopamine neurons signal phasic and ramping reward prediction error during goal-directed navigation

**DOI:** 10.1016/j.celrep.2022.111470

**Published:** 2022-10-11

**Authors:** Karolina Farrell, Armin Lak, Aman B. Saleem

**Affiliations:** 1 Institute of Behavioural Neuroscience, University College London, London WC1H 0AP, UK; 2 Department of Physiology, Anatomy and Genetics, University of Oxford, Oxford OX1 3PT, UK

**Keywords:** dopamine, VTA, Q-learning, miniscope, goal-directed, navigation, virtual reality, reinforcement learning, TD error, ramping

## Abstract

Goal-directed navigation requires learning to accurately estimate location and select optimal actions in each location. Midbrain dopamine neurons are involved in reward value learning and have been linked to reward location learning. They are therefore ideally placed to provide teaching signals for goal-directed navigation. By imaging dopamine neural activity as mice learned to actively navigate a closed-loop virtual reality corridor to obtain reward, we observe phasic and pre-reward ramping dopamine activity, which are modulated by learning stage and task engagement. A Q-learning model incorporating position inference recapitulates our results, displaying prediction errors resembling phasic and ramping dopamine neural activity. The model predicts that ramping is followed by improved task performance, which we confirm in our experimental data, indicating that the dopamine ramp may have a teaching effect. Our results suggest that midbrain dopamine neurons encode phasic and ramping reward prediction error signals to improve goal-directed navigation.

## Highlights

• VTA dopamine neurons have phasic and ramping activity during goal-directed navigation
• Pre-reward ramping is modulated by learning and task engagement
• A Q-learning model emulates phasic and ramping dopamine neuron activity in TD error
• Pre-reward ramps in dopamine neuron activity and TD error improve task performance

## Graphical abstract

**Figure:**
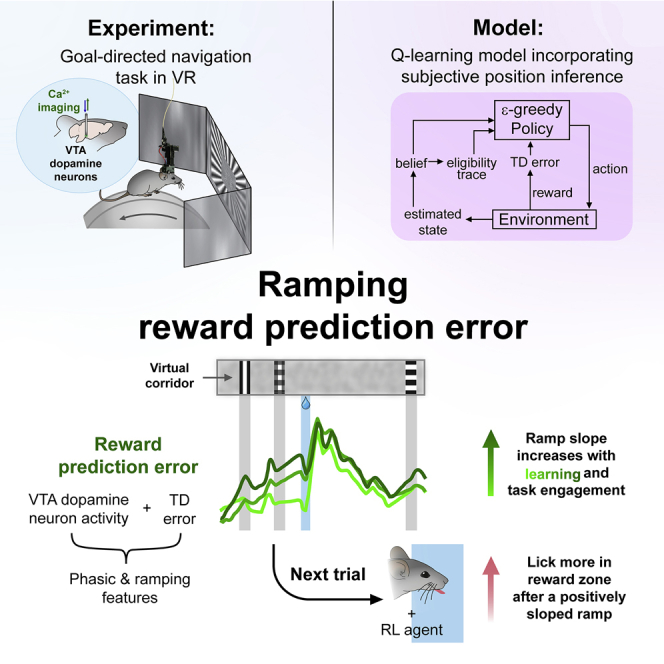

## Teaser

Dopamine neurons signal reward prediction error in their phasic activity, but reports of ramping activity and its potential function remain controversial. Using experimental and computational methods, Farrell et al. show that dopamine neurons display phasic and ramping activity and both act as reward prediction error signals for improving goal-directed navigation.

## Introduction

In naturalistic environments, animals navigate in order to harvest rewards. Successful goal-directed navigation requires learning to accurately estimate location and select optimal actions in each location. Midbrain dopamine neurons are known to play a key role in reward value learning by encoding temporal difference (TD) error in their phasic activity (Schultz et al., 1997; Bayer and Glimcher, 2005; Cohen et al., 2012; Kim et al., 2012; Steinberg et al., 2013; Stauffer et al., 2016; Lak et al., 2016, 2020; Parker et al., 2016; Sharpe et al., 2017; Coddington and Dudman, 2018; Mohebi et al., 2019). While the majority of studies on dopamine have been performed in non-navigation contexts, several lines of evidence indicate that dopamine neurons could play important roles in navigation. For example, it is established that midbrain dopamine neurons play causal roles in producing place preference (Tsai et al., 2009; Stamatakis et al., 2013) and that dopamine neurons enhance spatial memory through their effects on hippocampal plasticity, place fields, and ensemble reactivation (Martig and Mizumori, 2011; Ghanbarian and Motamedi, 2013; McNamara et al., 2014; Rosen et al., 2015; Gomperts et al., 2015).

Recently, studies of freely moving animals have observed dopamine ramps as the animal progresses toward the reward location (Collins et al., 2016; Gao et al., 2021; Guru et al., 2020; Hamid et al., 2016; Howe et al., 2013; Kremer et al., 2020; Mohebi et al., 2019; Phillips et al., 2003; Roitman et al., 2004; Syed et al., 2016). In these studies, ramping dopamine has been interpreted as tracking value, goal proximity, or motivation. However, in freely moving animals, it is difficult to precisely define the stimuli that animals use for navigation or when they are attended to, making it difficult to examine how they might be encoded in neuronal activity. Virtual reality (VR) overcomes this limitation, as it allows for control over visual stimuli observed by animals during navigation. A recent study using “teleports” in a virtual corridor proposed that pre-reward dopamine ramping reflected reward prediction error as opposed to value (Kim et al., 2020). However, this study did not necessitate goal-directed navigation, as animals did not need to learn the reward location and actively report it.

A similar VR experiment requiring animal locomotion toward reward showed that a subset of dopamine neurons displayed pre-reward ramping (Engelhard et al., 2019), although they did not explore the development or function of these ramps, limiting their interpretation of the ramps to encoding of spatial position in fully trained mice. It is therefore unknown whether dopamine ramps reflect reward prediction error during goal-directed navigation, where progression toward the goal is dependent on the animal’s actions, how these ramps arise, and what their functional role is in the learning and performance of goal-directed navigation tasks.

To address this, we imaged dopamine neural activity longitudinally as head-fixed mice learned to perform a goal-directed navigation task in closed-loop VR. This required animals to locomote to proceed through the corridor and estimate the location of a hidden reward zone. The task also differentiated between when the animal was actively engaged in finding the reward location or not, allowing a comparison between active navigation and passive viewing of VR.

Across learning, phasic dopamine responses developed that resembled reward prediction errors and indicated the animal’s estimate of the reward location. We also observed the development of pre-reward ramping activity, the slope of which was modulated by both learning stage and task engagement. The slope of the ramp was correlated with the accuracy of reward estimation in the next trial, suggesting that the ramp played a teaching role in the selection of accurate location-specific action during navigation. We further devised a Q-learning model incorporating belief state inference, which could simultaneously produce phasic and ramping TD error, matching the dopamine neural activity recorded in the task. Our results indicate that midbrain dopamine neurons, through both their phasic and ramping activity, encode reward prediction error, which may provide teaching signals for goal-directed navigation.

## Results

### Mice perform goal-directed navigation in VR

**Figure 1:**
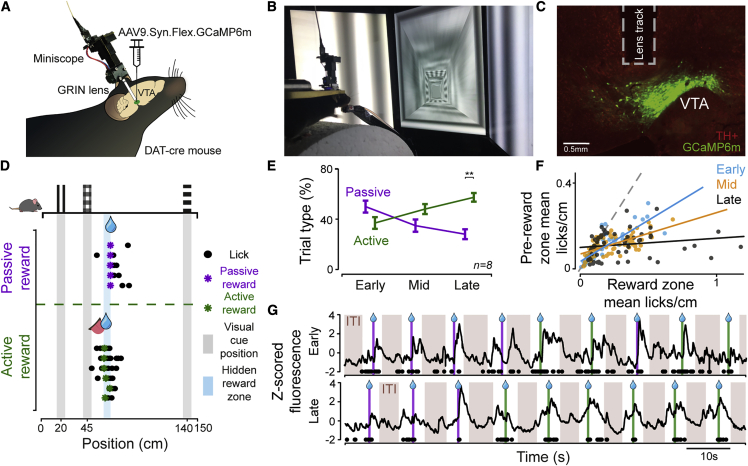
Mice learn to navigate in virtual reality and report the reward location (A) DAT-cre mice were injected with AAV9.Syn.Flex.GCaMP6m in the VTA and implanted with a GRIN lens over the VTA for imaging dopamine activity. (B) Head-restrained mice performed a navigation task by running on a cylindrical treadmill and virtual corridor displayed on three screens. (C) Example histological image showing GCaMP6m expression (green) in VTA TH+ neurons (red) and lens track. (D) Example behavioral performance shown on a schematic of the corridor with the position of the cues and reward zone, with the licks (circles) and reward delivery (asterisks) on example trials shown in rows. Licking within reward zone results in active reward delivery (green), and not licking within reward zone results in passive reward delivery (purple). (E) Mean percentage of passive and active trials across training stages (late stage, p = 0.0078, n = 8 animals, Mann-Whitney U test). Error bars indicate standard error. (F) Comparison of pre-reward lick rate in the reward zone (60–67 cm) versus pre-reward zone (50–59 cm) at different training stages. (G) Global fluorescence over several trials from a single animal from early-stage (top) and late-stage (bottom) training stages. Passive (purple) and active (green) reward deliveries (lines), licks (black circles), and intertrial intervals (ITIs; brown shaded regions) are indicated along the timeline. See also Figure S2.

To examine the activity of midbrain dopamine neurons during goal-directed navigation, we designed a task in a VR corridor. Head-restrained mice of both sexes were free to self-pace their locomotion on a treadmill, which accordingly updated visual scenes in a closed-loop system (Saleem et al., 2018) (Figure 1B; Video S1). A specific region of the virtual corridor had a hidden reward zone, where a lick triggered the delivery of sweetened water (Figure 1D). The reward zone was not explicitly marked by cues, and therefore the mice had to learn to estimate the location based on visual cues passed in the corridor and their own locomotion. If mice licked within the reward zone, they actively triggered reward delivery (active trial), whereas if mice did not lick in the reward zone, reward was delivered at the end of the reward zone (passive trial). Active trials indicated that the mouse had learned the reward location and reported their subjective estimate of it by licking accurately within the reward zone. We assessed the effects of learning by dividing training sessions into three stages: “early,” “mid,” and “late” per animal (see STAR methods). We found that mice performed more active trials and fewer passive trials with increased training (Figure 1E; n = 8, p = 0.0078, Mann-Whitney U test; see Table S1), consistent with them learning the location of the reward zone. Early in training, passive trials indicate that the animal has not yet learned the reward location, whereas later in training, they may indicate task disengagement, erroneous estimation of reward location, or attentional lapses. Mice also increased their licking frequency in the reward zone over training (Figures 1F and S2), indicating that their estimation of the reward location improves over training and that they successfully learn to perform the goal-directed navigation task.

Video S1. Video of mouse behavior, related to STAR Methods Video showing experimental setup and the behaviour of a mouse over two trials of the goal-directed navigation task in virtual reality. The mouse was head-fixed over a cylindrical treadmill with a Miniscope attached to its head. The mouse ran at a self-defined pace through a virtual corridor displayed on three screens, and licks in the lick port to receive reward. Reward delivery is heard as a click as the reward valve opens. The video is annotated to visually show when the reward is delivered (blue dot). After the corridor has been traversed, the screens display iso-luminant grey for an intertrial interval (4-6s), after which the animal is transported back to the start of the corridor to initiate the next trial.

As mice learned to perform the task, we measured the global activity of midbrain dopamine neurons. We expressed a genetically encoded calcium indicator (GCaMP6m; Chen et al., 2013) using viral transfection in the ventral tegmental area (VTA) of DAT-cre transgenic mice. We implanted a GRIN lens above the VTA and measured global calcium indicator fluorescence using a Miniscope (Ghosh et al., 2011) (Figures 1 and S1). We observed robust phasic responses that followed the reward delivery in individual trials (Figure 1G). Early in learning, dopamine responses mainly appeared after the reward delivery, while later in learning, we observed elevated activity both prior to as well as following the reward delivery, consistent with previous studies (Bayer and Glimcher, 2005; Coddington and Dudman, 2018; Cohen et al., 2012; Kim et al., 2012; Lak et al., 2016, 2020; Mohebi et al., 2019; Parker et al., 2016; Schultz, 2015; Schultz et al., 1997; Sharpe et al., 2017; Stauffer et al., 2016; Steinberg et al., 2013).

### Phasic reward prediction error coding in VTA dopamine neuron activity

**Figure 2:**
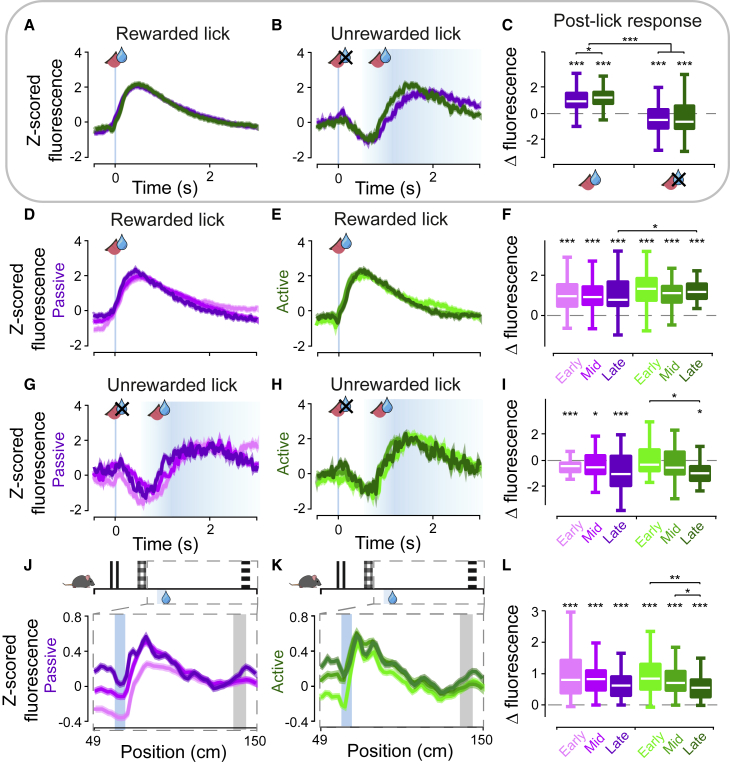
Phasic VTA dopamine activity reflects reward prediction errors (A and B) VTA dopaminergic activity as a function of time following rewarded (A) and unrewarded (B) licks for passive (purple) and active (green) trials, with SEM shown by semi-transparent areas. Rewarded licks were taken from trials with no licks prior to reward, and the aligned lick is the first lick following reward delivery. Unrewarded licks were taken from trials with one lick >0.5 s prior to reward delivery. (C) Boxplots of change in fluorescence following rewarded (left) and unrewarded (right) licks, measured as maximum difference in the window of 0–0.6 s following the lick. Boxplots indicate median across recording sessions (white), 25^th^ and 75^th^ percentiles as edges, and whiskers indicate most extreme points (outliers not shown). Asterisks directly above boxplots indicate significant difference from zero when Bonferroni corrections are applied such that ^∗∗∗^p < 0.0003, ^∗∗^p < 0.0033, ^∗^p < 0.0167; Wilcoxon signed rank test, see Table S1). LMM analysis indicated that rewarded versus unrewarded condition is significant (Model4: p < 0.0001, b = −1.4713, 95% CI [−1.7371,−1.2056], t = −10.874). (D–I) Same as (A)–(C), split by training stage. Bonferroni corrections are applied to comparisons across training stages in I) such that ^∗∗∗^p < 0.0003, ^∗∗^p < 0.0033, ^∗^p < 0.0167. LMM analysis confirmed that neither trial type nor session had a significant effect on post-rewarded lick change in fluorescence but that session did have a significant effect on post-unrewarded lick change in fluorescence (Model1: p = 0.0020, b = −0.0305, 95% CI [−0.0497,−0.0112], t = −3.1205). (J–L) Mean dopamine activity as a function of position in the corridor, focused on 49–150 cm, with SEM shown by semi-transparent areas. Change in fluorescence in (L) is calculated as the maximum value in the reward window (60–90 cm) minus the mean value in the pre-reward window (50–60 cm; see Figure 3 for explanation of differences). Change in fluorescence decreases over learning (p < 0.05, Mann-Whitney U test, see Table S1). Bonferroni corrections are applied to (L). LMM analysis confirmed that session had a significant effect on the post-reward change in fluorescence (Model3: p = 0.0378, b = −0.0216, 95% CI [−0.0421,−0.00123], t = −2.08435, see Table S2). See also Figure S3.

Dopamine activity showed sharp, transient increases and decreases following rewarded and unrewarded licks, respectively (Figures 2A and 2B; see Figure S3A for example lick positions). We calculated the magnitude of phasic responses as the change in activity from the time of lick to the peak of the response (Figure S3D). Averaged across all sessions, rewarded licks had positive responses (Figure 2A; p < 0.0001, Wilcoxon signed rank test), which were larger in active trials compared with passive trials (Figure 2C; p = 0.0155, Wilcoxon signed rank test). In contrast, unrewarded licks just before reward delivery were followed by a transient suppression in activity (Figure 2B; p < 0.0001, Wilcoxon signed rank test). This suppression was followed by a positive phasic response later in the trial, when reward was eventually delivered. The magnitude of suppression was similar in both active and passive trials (Figure 2C) but different from responses following rewarded licks (p < 0.0001, Mann-Whitney U test and linear mixed modeling [LMM] Model4, see Table S2). The suppression was consistent with activity suppression we observed in trials where we omitted rewards late in training (Figures S3B and S3C). Licks far from the reward zone did not display such suppression (Figures S3H–S3J), indicating that the suppression was regulated by expectation of reward rather than resulting from the licking action itself.

We also examined how these phasic dopamine responses changed over learning (Figures 2D–2I). In the time axis, phasic activity following rewarded licks did not change significantly over learning (Figure 2F). For unrewarded licks, we saw the post-lick suppression increase across training in active trials (Figure 2I; early versus late stage: p = 0.0078, Mann-Whitney U test; see Table S1) but not in passive trials. These results are confirmed by LMM, which showed that neither trial type nor session had significant effects on post-rewarded lick change in fluorescence (p > 0.05), but there was a significant effect of session on post-unrewarded lick change in fluorescence (Model1: p = 0.0020, b = −0.0305, 95% confidence interval [CI] [−0.0497,−0.0112], t = −3.1205; see Table S2). Measured along corridor position (Figures 2J and 2K), we observed the magnitude of reward responses decrease over training in active trials (Figure 2L; e.g., early- versus late stage: p = 4.0522e−04, Mann-Whitney U test; LMM Model3: p =0.0378, b = −0.0216, 95% CI [−0.0421,−0.00123], t = −2.08435; see Tables S1 and S2). This reduction in magnitude was mainly reflected in the altered pre-reward activity, which we explore in more detail in the next section.

In summary, the learning-related changes in peri-lick phasic neural activity, particularly when examined in the spatial dimension, are broadly consistent with the reward prediction error term of TD reinforcement learning (RL) models (Sutton and Barto, 2018; Schultz et al., 1997). The activity suppression at the time of unrewarded lick close to the reward zone further implies that mice in this task have an expectation of reward at the time of lick, reflecting their subjective estimate of the reward location.

### Phasic cue responses and pre-reward ramping activity develop over training

**Figure 3:**
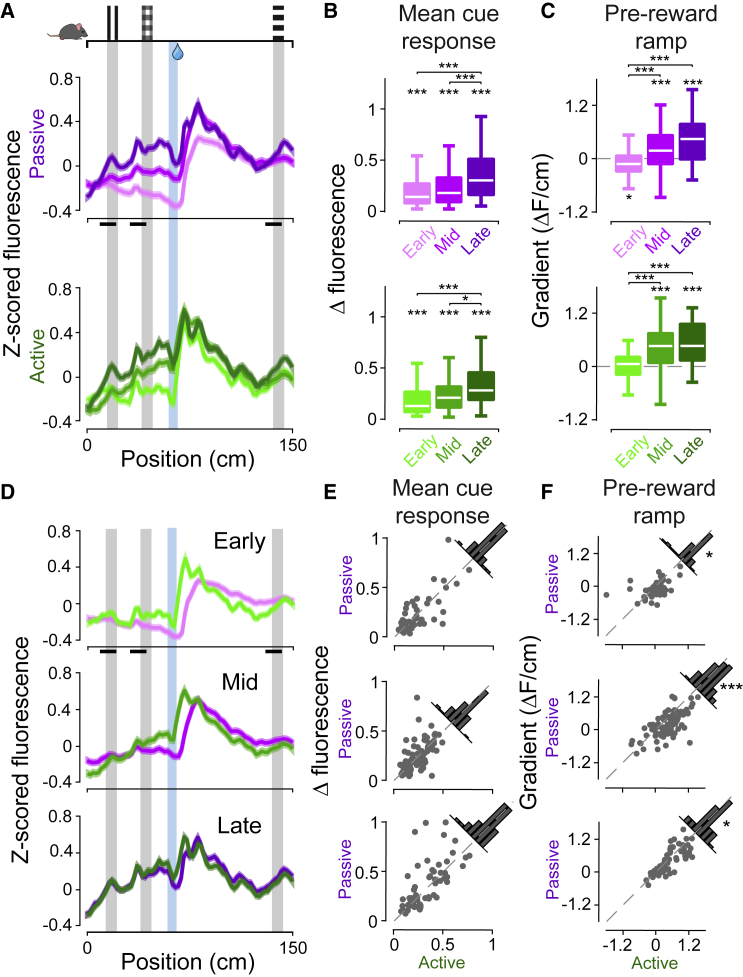
Phasic RPEs and slower pre-reward ramping dopamine activity develop over training (A) Activity as a function of position in the corridor, split into passive (top) and active (bottom) trials and different training stages, with SEM shown by semi-transparent areas. (B) Boxplots of the mean change in fluorescence in the cue windows indicated by the black bars in (A). All distributions are significantly larger than zero (p < 0.001, Wilcoxon signed rank test). Change in fluorescence increases over training (e.g., p = 1.0094e−05 for passive early-late, p = 5.3701e−05 for active early-late, Mann-Whitney U test, see Table S1). (C) Boxplots of pre-reward ramp gradient, calculated by fitting a line to activity in the 0–60-cm window. Median values for mid- and late-stage training are indicated above the white median line. Asterisks indicate distribution is significantly different from zero (p < 0.02, Wilcoxon signed rank test, see Table S1). Pre-reward ramp gradient increases over learning (e.g., p = 2.7530e−08 active early-late, Mann-Whitney U test, see Table S1). (D–F) Data shown in (A)–(C), directly comparing passive and active per training stage. Significant differences are found between active and passive ramp gradients at all training stages (Figure 4F, p = 0.0243, p < 0.0001, p = 0.0426, respectively, Wilcoxon signed rank test). See also Figure S4. LMM analysis confirmed that session had a significant effect on mean cue response (Model1: p < 0.001, b = 0.0083, 95% CI [0.00404,0.0125], t = 3.8491), while both trial type and session had significant effects on ramp gradient (Model2: p = 0.0146, b = −0.00206, 95% CI [−0.00371,−0.000408], t = −2.4529, and p < 0.0001, b = 0.00047, 95% CI [0.00028,0.00066], t = 4.8613, respectively, see Table S2).

Prior to reward delivery, we observed the development of phasic dopamine activity across training in response to reward-predictive cues, as well as a slow ramp in the pre-reward activity leading up to the reward zone location (Figure 3). Phasic dopamine responses to cues increased over training for both passive and active trials (Figure 3B; e.g., early versus late stage: p < 0.0001, Mann-Whitney U test). We next examined the slow ramp in pre-reward activity leading up to the reward zone location. Strikingly, we saw that the gradient of pre-reward ramping activity increased over learning in both passive and active trials (Figure 3C; early versus late stage: p < 0.0001, Mann-Whitney U test). We saw the same patterns of neural activity emerge over training in trials in which mice did not lick prior to the reward zone, indicating that pre-reward ramping activity was not caused by licks prior to the reward location (Figure S4).

As it has been suggested that increased dopamine activity could reflect increased motor vigor (Beierholm et al., 2013; Hamid et al., 2016; Ikemoto and Panksepp, 1999; Niv et al., 2007; Salamone and Correa, 2012; Salamone et al., 2003; da Silva et al., 2018), we also examined whether locomotor speed could explain the ramping activity. However, ramping dopamine activity did not reflect general locomotor vigor, as mice in our task generally slowed down on approach to the reward location, while dopamine activity ramped up instead (Figure S2). This resulted in the ramp gradient and change in speed leading up to the reward zone being anti-correlated or uncorrelated (Figure S2C). Overall, analysis of individual trials indicated that pre-reward ramping was not dependent on pre-reward slowing (Figure S2D).

To examine the effect of trial type, we compared cue responses and ramping activity between active and passive trials across the different training stages (Figure 3D). We found that the slope of the pre-reward ramp in active trials was larger than in passive trials at all training stages (Figure 3F; p = 0.0243, p < 0.0001, p = 0.0426 for early, mid, and late stages, respectively, Wilcoxon signed rank test), while mean cue responses remained similar (Figure 3E). This suggests that the gradient of the ramp was modulated by task engagement.

To confirm that both learning and task engagement impacted ramp gradient, we implemented LMM (see STAR methods) that either modeled the ramp gradient as a function of session or a function of session and trial type, with independent random effects terms for intercept and slope with animal identity as the grouping. The model including both session and trial type (Model2) had a significantly better goodness of fit compared with the reduced model (Model1) (p = 0.031033, Akaike information criterion [AIC] −2,781.8 [Model2] versus −2,779.1 [Model1], likelihood ratio test) where the fixed effects of trial type and session were both significant (Model2: p = 0.0146, b = −0.00206, 95% CI [−0.00371,−0.000408], t = −2.4529, and p < 0.0001, b = 0.00047, 95% CI [0.00028,0.00066], t = 4.8613, respectively; see Table S2). When the same models were applied for the mean cue response, we found that Model2 was no better than Model1 (p = 0.1719, AIC −470.01 [Model2] versus −470.49 [Model1], likelihood ratio test) and that Model1 showed a significant effect of session (p < 0.001, b = 0.0083, 95% CI [0.00404,0.0125], t = 3.8491). Together, thes LMM analyses matche the findings from non-parametric tests.

While ramping dopamine signals have been observed under certain conditions (Collins et al., 2016; Engelhard et al., 2019; Fiorillo et al., 2003; Gao et al., 2021; Guru et al., 2020; Hamid et al., 2016; Hamilos et al., 2021; Howe et al., 2013; Kim et al., 2020; Kremer et al., 2020; Mohebi et al., 2019; Phillips et al., 2003; Roitman et al., 2004; Syed et al., 2016; Wang et al., 2021), their functional role is yet to be agreed upon. Suggested functions include encoding of goal proximity (Engelhard et al., 2019; Guru et al., 2020; Howe et al., 2013), uncertainty (Fiorillo et al., 2003), goal-directed action encoding (Hamilos et al., 2021; Kremer et al., 2020; Syed et al., 2016), motivation or value (Collins et al., 2016; Gao et al., 2021; Hamid et al., 2016; Howe et al., 2013; Lloyd and Dayan, 2015; Mohebi et al., 2019; Niv et al., 2007; Wang et al., 2021), and reward prediction error (Kim et al., 2020). Given our observations that the gradient of ramping was modulated by learning and task engagement, in a similar way to phasic dopamine responses (Figures 2C and 2F), we hypothesized that the dopamine ramp might reflect reward prediction error (RPE). We therefore opted to test whether pre-reward ramping TD errors could be produced in an RL framework designed to match the strategies and performance of the animals in the behavioral task.

### Q-learning model recapitulates behavioral and neural data

As phasic cue and reward responses readily fit into a standard TD learning framework but ramping does not, we devised a model to investigate whether ramping could be explained as RPE. As Q-learning is a model-free algorithm that learns action values per state rather than state values (Sutton and Barto, 2018), we considered it more appropriate for use in a navigation context, where goal approach is dependent on selected actions.

**Figure 4:**
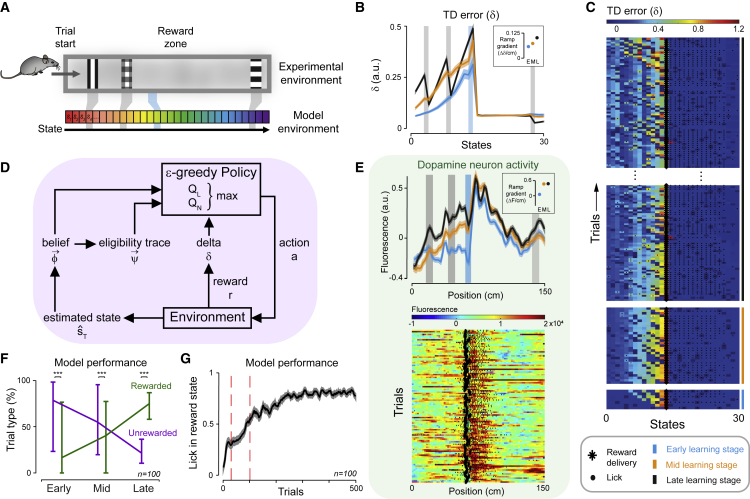
Model design and qualitative outputs (A) Relationship between experimental and model environments. (B) Mean of 100 agents’ δ over early, mid, and late training stages. Inset shows mean ramp gradient per learning stage (E, early; M, mid; L, late). (C) Example model run TD error (δ) output, showing trials where the agent licked in the reward state. Reward delivery is indicated with an asterisk, and licks are shown as black dots. (D) Summary of model architecture. (E) Top: mean calcium fluorescence for active trials from 8 animals for each training stage, with SEM shown by semi-transparent areas. Data taken from Figure 3A and recolored to match the model’s learning stages. Inset shows mean ramp gradient per training stage (see Figure 3C). Bottom: individual active trials from an example late-stage session from one animal. (F) Agents perform more rewarded (green) compared with unrewarded (purple) trials, i.e., licking in the reward state and receiving reward for each learning stage (Mann-Whitney U test). (G) Mean performance (licking in the reward state) smoothed over 10 trials across agents for the first 500 trials. Dashed red lines indicate the end of the early trials and mid trials, respectively. See also Figures S5–S7.

We devised a Q-learning algorithm that incorporated a position inference and an eligibility trace to simulate the animals’ learning in the task (i.e., where in the corridor to lick and where to refrain from licking) and the activity of their dopamine neurons (Figures 4, S5, and S6; for full details, see STAR methods). In designing the model, we opted for the simplest representations required to perform this task that could also reasonably be encoded by our mice during task performance. The model environment consisted of 30 discrete states, where each state simulates 5 cm of the VR corridor. In our experiment, mice have two information sources: visual (cues and optic flow) and self-motion, which we presume they use to inform their behavioral strategy. Cues can be readily incorporated into RL models using an eligibility trace (ψ) to keep a transient “memory” of the visual cues that passed. Position inference (based on visual and self-motion information) can then be incorporated via estimation of current state (${\hat{s}}_{T}$) (which can be noisy) and the construction of a “belief” distribution (${\vec{\varphi }}_{s}$) of current position relative to the environment, weighted by uncertainty related to the absence of nearby cues. Importantly, the peak of this distribution is taken as the “belief state” ($s_{B}$; see STAR methods), which is then used for subsequent updating. These two representations of position (${\vec{\varphi }}_{s}$) and cues (ψ) then weight the action values of licking ($Q_{L}$) or not licking ($Q_{N}$) at each state. Action selection is then performed by comparing $Q_{L}$ and $Q_{N}$, and an outcome (*r*) is received. The outcome is then used to calculate the prediction error (δ), which updates the value of the chosen action. The algorithm then iterates to the next state and repeats the process. For simplicity, we designed the algorithm such that if the agent chooses to lick in the reward state, it receives a reward value *r* of 1 but no reward if it does not lick. Similar to the behavioral task, we imposed a threshold of 2 licks prior to the reward state to prevent continuous licking, such that if the agent exceeded this threshold, it received a reward value of −0.1, and the trial terminated.

We ran 100 agents for 4,000 trials each and found that the agents learned to perform the task in a similar manner to our mice, slowly improving the ratio of rewarded to unrewarded trials over learning (Figures 4F and 4G). Over learning, the agents learned to have higher belief values $max\left(\vec{\varphi }\right)$, particularly when in the cue states (and neighboring states) compared with others (Figure S5A). Both the values of licking ($Q_{L}$) and not licking ($Q_{N}$) increasingly ramp prior to the reward state, with the value of licking spiking and the value of not licking plummeting at the reward state (Figures S5D and S5E). This competition between the two ramping action values can represent the need to inhibit an action until the correct location has been reached. TD error (δ) develops phasic spikes at pre-reward cue states over learning as well as a ramp over the pre-reward states that elevate over learning (Figures 4B and S6B). This is similar to the dopamine neuron activity recorded in our experiment (Figures 4E and S6A), with some differences in early-stage training. Specifically, TD error was seen to ramp, but not show cue responses, in the model in early-stage learning, while VTA dopamine neuron activity showed cue responses but little ramping. These differences may result from the high salience of visual cues in the behavioral task and faster learning of state information in the model compared with in mice, and we explore this further in our discussion.

TD error allows learning prior to reward delivery because it is calculated by comparing the values of consecutive states due to its method of bootstrapping from predictions of value of the current and subsequent states. In TD models, this TD error signal moves backwards to assign credit to reward-predictive stimuli, producing cue responses (Schultz et al., 1997). Why, then, do we see sustained ramping along the states that precede the reward location? Given the relationship between TD error and value in TD learning models, ramping TD error can occur when value also ramps (Gershman, 2014). In the model, both the Q-values for licking and not licking ($Q_{L}$ and $Q_{N}$) ramp across pre-reward states (Figure S5). To explain this, consider a trial where the model correctly believes it is in the reward state, chooses to lick, receives reward, and $Q_{L}$ is updated accordingly. On a future trial where the agent does not lick in a state (and $Q_{N}$ is still low at this point) but believes that the next state is the highly valued reward state (high $Q_{max}\left(s+1\right)$), TD error will be the discounted (but still large) difference between these two values ($\gamma \;Q_{max}\left(s+1\right)-Q_{N}\left(s\right)$), which is then also used to update $Q_{N}$. If, on the subsequent trial, the model similarly does not lick in the state that it believes is prior to one with high value, this value can propagate backwards across trials. With further trials, this increase in value can also bleed into $Q_{L}$. While this can lead to more licks prior to reward, a pre-reward lick threshold can help reduce pre-reward $Q_{L}$ with respect to $Q_{N}$, and therefore we include it to more closely match the conditions our animals experienced in the goal-directed navigation task. (If there is no lick threshold then this will not occur, but there is also a greater chance that they will proceed to the reward state and therefore will have more rewarded trials, so the backwards propagation of $Q_{N}$ is also facilitated [Figure S7B]).

The phasic cue responses in TD error can then be explained as a consequence of the reduction in uncertainty in position inference when a cue is passed. In the model, there is uncertainty of which state is being occupied, but this uncertainty is reduced when in the presence of a reward-predictive cue: these locations provide more certainty that this is not the state to lick in. Therefore, the estimated current value of not licking ($Q_{N}\left(s\right)$) in those states is increased, and when this is subtracted from the discounted $Q_{max}\left(s+1\right)$, it results in peaks in TD error for the states before the cue states.

In summary, the model recapitulates much of our experimental data, providing a theoretical explanation for why dopamine activity ramps during goal-directed navigation.

### Pre-reward ramp improves task performance on subsequent trial

**Figure 5:**
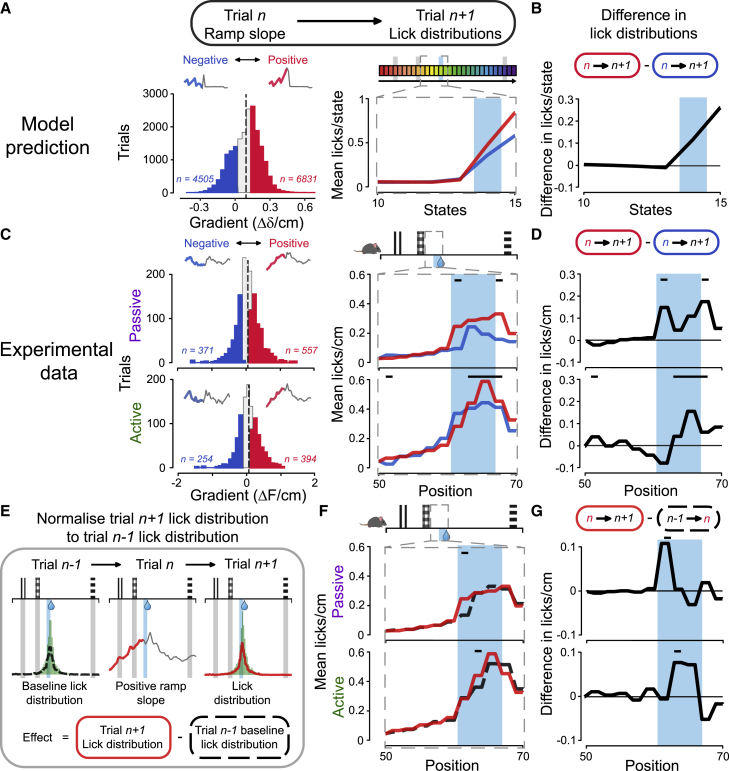
Positive dopamine ramp slope improves task performance on subsequent trial (A) Left: Q-learning model predicts a distribution of ramp slopes in trials late in learning that have no licks prior to the reward state (to avoid negative RPEs). The highest third of ramp slopes define the group of “positive slope” trials (red), and the lowest third below zero define the “negative slope” trials (blue). Intermediate trials are shown in gray, and the mean gradient across all trials is shown by the black dashed line. Inset: mean TD error trace for the grouped trials. Pre-reward is shown in red or blue, and the rest are shown in gray. Right: the mean licks per state of the trials following those indicated in the left panel (trial n + 1). The reward state is indicated in light blue. (B) The difference between the lick distributions shown in the right panel of (A). (C and D) The same analysis as in (A) and (B) but using experimental data from eight animals and licks per cm. Light blue bars indicate the reward zone. Black bars above the lick distributions indicate significant differences (p < 0.05, Mann-Whitney U test, see Table S1). (E) Schematic of trial-to-trial effect calculation, indicating how the lick distribution of trial n − 1 is used as a baseline for normalization of the positive ramp slope’s effect on the lick distribution for the subsequent trial. (F) Lick distributions for the trials preceding (black, dashed) and following (red) a positive ramp slope trial. (G) Difference between the lick distributions in (F). See also Figure S8.

Having established that ramping activity can be explained as prediction error in conjunction with classical phasic RPEs to cues and reward, we posited that this ramping prediction error should have a teaching function similar to phasic RPEs. On examination of individual trials in the model’s late-stage learning (where mean pre-reward TD error slope was maximal), we found a distribution of slopes (Figure 5A, left). We classed the highest third as a “positive slope” group and the lowest third below zero as a “negative slope” group and observed that the TD error traces for these groups both had phasic responses to cues and reward, but, as expected, only the positive slope group had a clear ramp prior to the reward state (Figure 5A, left inset).

The model predicted that a positive slope in pre-reward TD error (on trial n) should be followed by increased licking in the reward state on the subsequent trial (trial n + 1) compared with the negative slope trials (Figures 5A, right, and 5B). This can be explained as a result of a higher pre-reward ramping of $Q_{N}$ compared with $Q_{L}$, such that not licking is favored in pre-reward states, which facilitates reaching the reward state on that trial but also increases the likelihood that similar not-licking actions are chosen for the pre-reward states on the next trial. Given this model prediction, we therefore performed the same analysis on our experimental data. As in the model, we found distributions of pre-reward dopamine neuron activity ramp slopes that averaged to a positive gradient, in both passive and active trials. When we looked at the licking distributions on the subsequent trial, we saw the same effect: positive ramp slope trials are followed by increased licking in the reward zone compared with negative ramp slope trials, both in passive and active trials (Figures 5C and 5D; p < 0.05, Mann-Whitney U test). Interestingly, the exact positions in the reward zone where this effect was significant differ between active and passive trials and may reflect different learning needs to refine licking behavior. For example, passive trials indicate that the animal licked too late and missed the reward zone, so on the subsequent trial, the animal should lick earlier, at the start of the reward zone. Conversely, in active trials, the animal licked in the correct location, so the ramp slope effect reinforces licking in the center of the reward zone. To clarify that this effect was not the result of slow fluctuations in behavior across trials, we also normalized the lick distribution on trial n + 1 by subtracting the lick distribution of trial n − 1 and still saw a subtle but significant effect of the positive slope pre-reward ramp on reward zone licking (Figures 5E–5G). The same analysis for negative ramp slope trials did not yield significant effects on reward zone licking (p > 0.05 in both active and passive trials, across all reward zone bins tested), indicating that this effect was limited to positive pre-reward ramp slopes rather than ramping in general. Interestingly, the same analysis on the effects of reward response sizes on subsequent trials showed that a small reward response on trial n was followed by increased reward zone licking on trial n + 1 when trial n was active, but not passive, although this was not found to be significant (Figure S8). Together, these data and the model suggest that a positive pre-reward ramp slope is a form of RPE that may reflect a teaching signal to improve the accuracy and frequency of reward location reporting on the subsequent trial, serving to optimize behavior even in late-stage training.

## Discussion

Our results indicate that both phasic as well as slower ramping of dopamine activity may reflect RPE teaching signals that can improve the accuracy of goal-directed navigation. We observed the development of positive phasic responses to reward and reward-predictive cues and negative phasic responses following unrewarded licks. In addition, we observed a ramping of dopamine activity leading up to the reward location, the gradient of which was increased with learning and task engagement. We show that a Q-learning model could explain both phasic and ramping RPEs and could predict improved task performance following the pre-reward ramp, which we also saw in our experimental data.

Dopamine neurons are known to play a key role in learning by signaling RPE, as established in the temporal domain in tasks such as Pavlovian conditioning, and recapitulated in TD learning models (Sutton and Barto, 2018; Schultz et al., 1997). However, their activity has been less well studied in spatial tasks. This is in part due to the use of freely moving animals, where there is limited control over when the animal perceives features of the task, and precise readouts of location estimation. By using VR, we were able to create a navigation task with high temporal precision and a precise readout of the animal’s estimate of the reward location through licking (Fournier et al., 2020; Saleem et al., 2018). This allowed us to measure neural responses to precise events such as cues, rewards, and licks and establish the presence of RPEs during spatial learning.

Our implementation of VR also has the advantage of being closed loop and requiring active navigation. If progression through the virtual corridor was simply presented as a video of movement at a predefined speed, irrespective of the animal’s own movements (i.e., open loop), spatial encoding could not be differentiated from an equivalent passive approach temporal task (Kim et al., 2020). In contrast, our closed-loop task gives control of movement (and corresponding visual scenes) to the animal, simulating more naturalistic navigation. In addition, the requirement for the animal to report the hidden reward location ensures that the animal is actively navigating to a goal rather than passively running through an environment (Kim et al., 2020). We were therefore able to characterize neural activity as a function of spatial position, which revealed ramping activity of VTA dopaminergic activity along the corridor until the reward location, similar to dopamine signals observed in animals navigating real environments (Collins et al., 2016; Gao et al., 2021; Guru et al., 2020; Hamid et al., 2016; Howe et al., 2013; Kremer et al., 2020; Mohebi et al., 2019; Phillips et al., 2003; Roitman et al., 2004; Syed et al., 2016).

We observed two patterns of activity: phasic and ramping. The observed patterns of phasic responses to reward-predictive cues and reward delivery were to be expected, as shown in many previous experiments (Bayer and Glimcher, 2005; Coddington and Dudman, 2018; Cohen et al., 2012; Kim et al., 2012; Lak et al., 2016, 2020; Mohebi et al., 2019; Parker et al., 2016; Schultz et al., 1997; Sharpe et al., 2017; Stauffer et al., 2016; Steinberg et al., 2013) and predicted by TD learning models (Sutton and Barto, 2018; Schultz et al., 1997). It has further been shown that phasic RPE dopamine signals are modulated by inferred belief state, such as subjective estimates of sensory signals (Lak et al., 2017) or the timing of reward delivery (Starkweather et al., 2017). Our results showing that RPE follows rewarded and unrewarded licks suggest that phasic dopamine signals can also represent belief about estimated reward location given the visual and self-motion information available in our navigation task.

Pre-reward ramping dopamine has been observed in many studies (Collins et al., 2016; Engelhard et al., 2019; Fiorillo et al., 2003; Gao et al., 2021; Guru et al., 2020; Hamid et al., 2016; Hamilos et al., 2021; Howe et al., 2013; Kim et al., 2020; Kremer et al., 2020; Mohebi et al., 2019; Phillips et al., 2003; Roitman et al., 2004; Syed et al., 2016; Wang et al., 2021) but without a clear consensus on its function or whether it originates in dopamine neuron activity or is an epiphenomenon in downstream striatum as a result of synaptic modulation. We find that pre-reward ramping is observable in the global calcium imaging of dopamine neurons in the VTA even in head-fixed, goal-directed navigation. Given that dopamine ramping appears fixed to the reward location, as shown in freely moving navigation (Howe et al., 2013), we inferred that the ramp might convey a spatially relevant signal, similar to a successor representation place field tied to reward position (Sosa and Giocomo, 2021; Stachenfeld et al., 2017). We find that the ramp develops and increases in slope over learning and also has a consistently greater slope during trials where the animal is actively engaged in reporting the reward location as opposed to when they are more disengaged. These characterizations are similarly applied to phasic RPEs (Lak et al., 2016; Parker et al., 2016; Schultz et al., 1997; Tanaka et al., 2019).

The finding that the ramp slope increases across learning has been observed previously in a fixed-distance locomotor task (Guru et al., 2020), and this feature appears to depend on the nature of the task at hand (or the strategy required), as ramping ceases to exist with extended training in other tasks. From similar studies, it can be observed that dynamic sensory cues that are indicative of goal proximity appear to be sufficient to induce ramping prior to “distant” rewards (Kim et al., 2020) (although they are not required [Gao et al., 2021]), but ramping seems to only persist in tasks that require some form of internal model or ongoing computation for adequate behavioral performance (Guru et al., 2020). For example, in tasks that require reward approach in the absence of clear landmark cues (as in our task; Collins et al., 2016; Guru et al., 2020), the ramp persists across learning. In tasks where a strategy can be learned such that a particular cue is sufficient to fully predict reward delivery with certainty and without requiring strategic action on the part of the animal, a pre-reward ramp can fade with extended training (Guru et al., 2020) (and this could explain apparently conflicting results regarding the relationship between ramping and action initiation [Gao et al., 2021; Syed et al., 2016]) In this respect, our results match with the conclusions of Guru and colleagues (Guru et al., 2020): that ramps persist when ongoing within-trial calculations are required for task performance. One difference between our results and those of Guru and colleagues’ fixed distance task (Guru et al., 2020) is that in our task we find that ramp slope actually increases rather than just persists, which could reflect the increased precision required in state estimation and action selection (i.e., licking within the 6.5-cm virtual reward zone compared with stopping wheel movement and going to the reward port after 5–9 turns [Guru et al., 2020], a comparatively larger and less precise target).

Our model can further explain why ramping occurs in different tasks with different time courses and gradients: goal-directed navigation necessitates state estimation under conditions of uncertainty and therefore produces ramping TD error. In some tasks, this uncertainty may be reduced so that ramps decay with extended learning (Guru et al., 2020) (Figures S7D and S7E), for example if the animal learns that a cue fully predicts reward delivery or develops habitual responding to minimize mental computation (Syed et al., 2016; Guru et al., 2020). However, other conditions may require trial-by-trial state estimation (e.g., spatial or internal state) (Gao et al., 2021; Guru et al., 2020; Wang et al., 2021) where uncertainty cannot be reduced and therefore the ramps persist. This may correspond with the proposal from Guru and colleagues that ramps may reflect the use of an internal model (Guru et al., 2020). Our model can therefore explain the heterogeneous ramps observed in previous studies: the ramping of TD error depends on the behavioral strategy required by the task at hand (Figure S7).

Finally, while dopamine has been suggested to play a role in vigor and motivation (Collins et al., 2016; Hamid et al., 2016; Howe et al., 2013; Lloyd and Dayan, 2015), we find that this ramp is unrelated to licking prior to the reward zone (Figure S4) and is inversely correlated or uncorrelated with speed (Figure S2), suggesting that it does not reflect action vigor (although this does not refute the possibility of the ramp encoding cognitive effort [Westbrook and Braver, 2016] or the specific goal-directed motivation to slow down in a speed-accuracy trade-off).

Given the similarities in the characterization of phasic and ramping signals and that the ramp did not reflect increased locomotor vigor, we asked whether the ramp could represent RPE using a Q-learning model. The model was given the basic information required to navigate to reward: a representation of self-location and a representation of visual cues observed. While simplistic, our model nevertheless recapitulated our experimental data, capturing how mice learned to lick in the reward location but not lick prior to that. The model reproduced both pre-reward ramping and also phasic responses to reward-predictive cues and reward delivery, as we saw in our calcium imaging data. Moreover, the model predicted that a positive pre-reward slope would be followed by increased reward location licking on the subsequent trial, which we also found in our experimental data. Together, this supports the idea that pre-reward ramping is a form of RPE that may provide a teaching effect to improve goal-directed navigation. To caveat this, the model was not designed to explain the difference between active and passive trials but rather to explain how a pre-reward ramp in TD error could arise.

### Limitations of the study

Firstly, our neurophysiological results are based on observation of global dopamine neuron activity, averaged across the entire field of view of neurons. Single-cell resolution could provide further information about whether ramping activity is a feature of all or subsets of neurons, as suggested by previous studies (Engelhard et al., 2019; Kremer et al., 2020).

Secondly, the Q-learning model presented is optimized for the behavioral task that our animals performed and would likely have to be altered to capture particular features of different tasks. Specifically, we used selective weighting of the two Q-values by the belief of being in the reward state and the eligibility trace, respectively. A more generalizable model would allow the weighting to come about over training rather than being hard coded. Another aspect is that we impose a negative reward value for the model if the pre-reward licking threshold is exceeded, whereas the mice just have the reward opportunity loss when the trial terminates. The inclusion of a small negative reward value was simplistic and allowed the model to learn that trial termination was a negative outcome to speed learning (Figure S7), although a more sophisticated model could instead use a state transition cost function to provide a similar reward opportunity loss to the agent when a trial terminated to parallel the opportunity cost the mice experience.

Finally, while the model mostly recapitulates our experimental data, there is a difference in VTA dopamine neuron activity and TD error in the early learning stage, where the model predicts ramping TD error, but our data instead show cue responses with minimal or no pre-reward ramping. This may be due to dopamine neurons responding to salient visual features, a feature that is reported in animals (Cai et al., 2020; Horvitz et al., 1997; Ljungberg et al., 1992; Menegas et al., 2018; Takahashi et al., 2017) but that is not accounted for in our Q-learning model, but could also reflect the differential timescales of learning processes, such that dopamine neurons place heavier weighting on discrete salient cues in an environment compared with learning the sequence of pre-reward states that are less easily delimited and identified in a real environment compared with a model one. The other discrepancy between the simulated TD error and our data is that in our imaging data, there appears to be a response to the final, non-reward predicting cue toward the end of the corridor. We were surprised to find this phasic activity just before the end of the corridor in our data, but we note two possible explanations: the response could reflect the upcoming opportunity for reward in the form of predicting the next trial, thereby reflecting a cross-trial cue response, or the response could simply reflect the salient end of the corridor and transition to gray screen that the mouse is approaching.

### Conclusions

From our results, we find substantial evidence that ramping dopamine encodes RPE. As many studies that have observed dopamine ramping have done so in tasks that require animals to navigate to goals, we posit that ramping might result from a need to integrate spatial and temporal information to perform the correct actions in the correct locations to obtain reward. This ramping further matches the characterizations and functions of RPE given the development over learning, the dependence on task engagement, and improved performance on the subsequent trial.

Overall, our data show a ramping of VTA dopamine neuron activity that is most consistent with encoding of RPE. The model unifies both phasic and ramping activity as a single RPE signal, and our analysis indicates the potential teaching effect of dopamine ramping signals in improving reward localization. Together, we conclude that VTA dopamine neurons, through both their phasic and slower ramping activity, signal RPE during the learning of goal-directed navigation.

## STAR★Methods

### Key resources table

**Table:** 

REAGENT or RESOURCE	SOURCE	IDENTIFIER
**Antibodies**		

Mouse anti-TH	ImmunoStar	Cat # 22941; RRID: AB_572268
Alexa Fluor 594 Goat anti-mouse	BioLegend	Cat # 405326; RRID: AB_2563308

**Bacterial and virus strains**		

AAV9-Syn-FLEX-GCaMP6m	Addgene	#100838; RRID: Addgene_100838

**Experimental models: Organisms/strains**		

Mouse: DAT-IRES-cre (Slc6a3^tm1.1(cre)Bkmn^)	The Jackson Laboratory	JAX006660; RRID: IMSR_JAX:006660
Mouse: C57BL/6	Charles River Laboratories	Strain code: 027; RRID: IMSR_CRL:027

**Software and algorithms**		

Bonsai	Lopes et al., 2015	https://bonsai-rx.org/; RRID: SCR_017218
UCLA Miniscope Bonsai node	Jonathan Newman	https://github.com/jonnew/Bonsai.Miniscope
MATLAB 2018a	MathWorks	https://www.mathworks.com/; RRID: SCR_001622
Custom MATLAB VR code	Saleem et al., 2013; Saleem et al., 2018	https://github.com/amansaleem/SaleemLab-VRhttps://doi.org/10.5281/zenodo.7074768
LAS X (Leica Application Suite) confocal microscopy software	Leica Microsystems	https://www.leica-microsystems.com/; RRID: SCR_013673

### Resource availability

#### Lead contact

Further information and requests for resources and reagents should be directed to and will be fulfilled by the lead contact, Dr Karolina Farrell (karolinajhfarrell@gmail.com).

#### Materials availability

This study did not generate new unique reagents.

### Experimental model and subject details

#### Mouse line creation and maintenance

The DAT-cre transgenic mouse line was started by breeding one male DAT-IRES-cre ($Slc6a3^{tm1.1\left(cre\right)Bkmn}$) mice (JAX006660, *The Jackson Laboratory*) with a female C57BL6 mouse (*Charles River*, Strain 027). Following genotypic identification (*Transnetyx*) of DAT-cre offspring, heterozygous DAT-cre breeders were selected and subsequently paired with C57BL6 breeders in order to maintain the colony. Following pregnancy confirmation, males were separated out. Pups were weaned three weeks after birth, earmarked for genotyping, and group-housed in single-sex cages. Mice were 8–16 weeks old at the start of behavioural training.

All mice were given environmental enrichment, standard chow and water *ad libitum* prior to the experiment. Mice were housed in a colony room at 21.5°C, 45% humidity on a 12 h/12 h light/dark cycle. Selected experimental mice were single-housed and underwent implant and baseplating surgeries. Following at least 7 days recovery, water-restriction was initiated to increase motivation, with free access to water overnight once every two weeks. Mice were weighed each day, received HydroGel® (*ClearH2O*) in their home cage following behavioural training to ensure sufficient hydration (>40 mL/kg), and had free access to standard chow to maintain their weight between 85–90% of their predicted unrestricted weight. Data from eight mice are presented (5 female, 3 male). Analysis of sex differences was not investigated as the sample size was insufficient for meaningful analysis.

### Method details

#### Surgeries

Experimental mice underwent two surgeries. In the first surgery, mice were induced with 3% isoflurane and maintained at 1.5%. Eye moisture and body temperature were maintained, analgesia (5% carprofen) was administered, and the head was shaved. In six animals, 5 mg/kg of 2% w/v dexamethasone was administered intramuscularly to reduce inflammation and brain swelling. Surgery was performed on a heated pad to maintain body temperature at 37°C. A craniotomy was performed directly over the VTA of one hemisphere (2 left, 6 right). 600 nL of AAV9-Syn-FLEX-GCaMP6m (*Addgene* Plasmid #100838) diluted 1:3 in aCSF was injected at a rate of 50 nL/min into the VTA (AP -3 mm, ML 0.5 mm, DV -4.6 mm from dura) and the pipette was left in place for 10 minutes. Following this, for six of the mice, a blunt needle was inserted and lowered between 1.5 and 2 mm from dura before being removed. The GRIN lens (*Inscopix* 1050-002179) was then inserted, at an approximate rate of 400–500 μm/min to a depth around −4.3 mm and secured in place using dental cement (Super-Bond C&B, *Sun Medical*). A custom metal headplate was cemented behind the lens, and a plastic cap (cut-off end of Eppendorf® tube) was cemented over the lens for protection. 0.2 mL of warmed saline was administered subcutaneously per hour of surgery to maintain hydration. Following recovery, mice were closely monitored and given 20 μL meloxicam in condensed milk and high-protein wet food for 3 days post-surgery.

The second surgery was performed 2–3 weeks after the first, to allow for viral expression and inflammation reduction. The mouse was similarly induced, maintained and monitored. Following head-fixation, the protective cap was drilled out and the lens was cleaned. A modified UCLA Miniscope (Ghosh et al., 2011) with an incorporated GRIN lens and with an attached baseplate was lowered to around 100–300 μm above the implanted lens, and the field of view explored using Bonsai software (Lopes et al., 2015) and the UCLA Miniscope node (see key resources table). When the optimal field of view was found, the baseplate was carefully cemented to the skull over the implanted lens. The Miniscope was removed and a protective Delrin cap (*S. Stiteler*, *miniscope.org*) was secured to the baseplate using a set screw.

#### Behavioural training

All behavioural training was performed during the dark cycle, and in a dark room. Mice were handled, water-restricted, and acclimatised to head-fixation on a custom Styrofoam wheel (Saleem et al., 2018) and Miniscope attachment for a few days prior to behavioural testing. Mice were also offered rewards (∼1.5-2 μL cherry-flavoured Kool-Aid, *Kraft Foods*), pseudo-randomly delivered, through a lick spout to encourage running and identify putative dopamine reward responses, while monitoring licks using a custom infrared sensor. Mice were free to run in the task as much as they desired for about 30 minutes during the dark cycle each day (∼5 days/week) on a custom rig, where they were presented with a virtual corridor on three screens (Figure 1B and Video S1). The three 9.7” screens (LP097QX1-SPAV with 4:3 aspect ratio, controlled by HDMI driver boards) were fixed in portrait mode at 120° from each other, such that they formed half a hexagon, and the mouse was placed at the centre of the hexagon. The mouse’s movements on the wheel were yoked to the visual display using a rotary encoder such that they could only navigate towards the end of the virtual corridor by moving in a forward direction (closed-loop system) (Saleem et al., 2013, 2018) (see key resources table). The rotary encoder, infrared lick detector, and reward valve (225P011-21, *NResearch*, USA) interfaced with the VR code through an Arduino Leonardo board. The task used a 150-cm long corridor, with a low-contrast white noise pattern along the ceiling, walls and floor (8-cm width and height). The visibility of the corridor was limited to 70 cm ahead. A full traversal through the corridor is considered a completed trial. Reaching the end (or timing out) initiated an ITI where the corridor was replaced with isoluminant grey. The ITI was chosen randomly between 4 and 6s, to ensure that timing between spatial features could not carry past each trial.

As the mice travelled down the corridor, they would pass two distinct patterned cues (8-cm wide) on the walls, centred at 20 cm and 45 cm along the corridor respectively. An unmarked reward zone spanned 60.5 cm to 67 cm in the corridor. On each trial, a reward was delivered to a spout in front of them. The spout incorporated an infrared sensor to detect licking. The exact location of the reward zone was not indicated by any cue and instead had to be estimated by the mouse based on prior cues and actions. If the mouse did not lick in the reward zone, then it would passively receive the reward at the end of the zone (passive trials). However, if it licked within the zone, then reward delivery was actively triggered (active trials), and therefore delivered earlier than in the passive trials (Figure 1D). The delay from the triggering lick to reward delivery was short but could allow for multiple licks to occur in the reward zone in quick succession prior to reward delivery in active trials. When reward was delivered, an audible click (muffled to reduce salience) could be heard as the solenoid valve opened (Video S1). The mouse could then continue down the virtual corridor and pass a final, non-reward-predictive patterned cue (centred at 140 cm) before the end of the corridor was reached (grey screen). If the mouse did not reach the end of the corridor within 30 seconds, the trial was terminated (timed out). A pre-reward licking threshold was also imposed to reduce licking and indicate the mouse’s estimation of the reward location. This was gradually reduced over training (following the mouse’s natural inhibition of excessive licking in incorrect locations) to approximately 8–10 licks in late-stage training. If the mouse exceeded this threshold prior to reward delivery, the trial was terminated.

For comparison of data across training, training sessions were split into three stages: early, mid and late training. Early and late stages were defined as the first and last quartile of sessions respectively for each animal, with the rest being classified as mid-training.

#### Calcium imaging

Calcium fluorescence was detected using a Miniscope through an implanted GRIN lens (Figure 1A) which acted as a proxy for dopamine neuron activity. A custom Bonsai workflow (Lopes et al., 2015) and Miniscope node (see key resources table) were used to acquire images (at 15 Hz) and calculate global calcium signal. Mice had a mean of 24 training sessions over the course of the experiment. In early to mid-training, active trials were incentivised by offering slightly larger rewards (∼2-3 μL) until the mice demonstrated the ability to repeatedly perform active trials (as judged by the experimenter), at which point the active trial reward volume was decreased to be the same as the passive trial reward volume. Later in training, 6 of the 8 mice had reward omission trials introduced pseudo-randomly in 5–7% of the trials in each session, where no reward was delivered but the mouse still traversed the corridor.

#### Data preprocessing

Imaging data collected using Bonsai was imported into Matlab R2018a (*MathWorks*, Natick, MA, USA) for pre-processing. Rarely, unstable signal was produced by Miniscope movement or power surges or lapses. Two types of signals were considered unstable. The first type was fluorescence that exceeded or fell below a threshold of 1.5 standard deviations away from the mean fluorescence across the whole training session. The second type was when fluorescence surrounding the first type (±100 ms) exceeded half of the difference between the maximal fluorescence and mean fluorescence, therefore constituting an ‘upswing’ or ‘downswing’ of a large transient. Following removal of these two types of unstable signal, a photobleaching curve was fitted across the entire session using Matlab polyfit (2^nd^ order) and subtracted from the fluorescence trace. The trace was then corrected for baseline variation by subtracting the lower 10% quantile baseline using a 60s window. The resulting signal was then aligned to the virtual corridor times. Trials with unstable signals were removed from subsequent analysis.

To be included in further analysis, trials, sessions and animals had to fulfil certain criteria. Aborted trials (time-outs, too many licks before reward or experimenter-terminated), and trials with unstable signal were excluded from analysis. Sessions were included if they had >50% trials with at least one lick, and >10% active trials. Four animals were excluded from further training as they did not show visible reward responses to random reward, and were later confirmed to have mistargeted GRIN lens placement. One animal was excluded due to a visual defect (cataract), and another one was excluded as it did not learn the task (based on having <50% of the sessions containing active trials). Subsequent analysis was performed on data that met these conditions (eight animals). Fluorescence was z-scored across each session. Figures 2 and 3 show data across sessions, Figure 5 shows data across trials from late-stage learning.

#### Data analysis

Behaviour during training was assessed through binned mean licks/cm across the virtual corridor, as well as through the speed of the wheel rotation. Phasic responses to the cues were calculated as the maximum minus the minimum values within each 12-cm cue window (10–22 cm, 32–44 cm and 132–144 cm respectively). Phasic responses to the reward were calculated as the maximum value in the reward window (60–90 cm) minus the mean of the activity within the pre-reward window (50–60 cm). Ramp gradient was calculated as the gradient of a fitted linear line (Matlab polyfit, 1^st^ order) to the fluorescence in the window 0–60 cm.

Rewarded lick traces (Figure 2) included only trials that did not have any licks before the reward zone, to avoid contamination of the signal by prior licks. Rewarded lick traces were also averaged across each animal before averaging over all animals to counter the appearance of an electrical artifact that was presented in two animals when reward was delivered during active trials. Unrewarded lick traces included only trials that had only one lick prior to the reward zone that was at least 0.5s before reward delivery. For comparison with reward omission trials, the data in Figures S3B and S3C only includes sessions that contained omission trials. Suppression gradient was calculated as the mean of the gradients of fitted lines (Matlab polyfit, 1^st^ order) between the fluorescence at zero and the minimum fluorescence in the second half of the window of fluorescence being examined (here −2.5 to 3s around the lick, so minimum value between 0.25 and 3s) for each trial. Paired data was tested for differences using the Mann-Whitney U test (Matlab ranksum), as were tests of difference from zero, while the Wilcoxon signed rank test (Matlab signrank) was used to test for differences between different training stages. These results were then confirmed using linear mixed modelling (Matlab fitlme), which accounts for repeated measures in our longitudinal experimental dataset.

Ramp slopes were classified as positive or negative by considering all ramp slopes across all trials, sessions and animals and taking the most positive third as ‘positive’ and the most negative third (below zero) as ‘negative’. For analysis of the effect of ramp slope on trial *n* on lick distribution on trial n+1, only trials with no licks before the reward zone were considered to eliminate any contamination of signal by licks. Experimental lick distributions in Figures 5C, 5D and 5F and 5G were calculated as licks/cm smoothed from 2 cm bins.

#### Histology

Mice were deeply anaesthetised using 3.5% isoflurane, injected with a lethal dose of pentobarbital (Euthatal, *Boehringer Ingelheim*) intraperitoneally, and transcardially perfused with 1X PBS followed by 10% formalin solution. Following perfusion, the brain was extracted and placed in 10% formalin for short-term storage. Prior to sectioning, the brain was placed into a 30% sucrose solution until it sank, for cryoprotection. The brain was then mounted upright in OCT (*Sakura* FineTek) and 40 μm slices were made using a cryostat (*Leica* CM1850 UV). Slices were washed five times in 1X PBS before overnight incubation on a rotating platform at room temperature in primary solution: 1:5000 mouse anti-TH (*ImmunoStar*, Cat #22941) in PBS-T (0.4% Triton in 1X PBS), to label TH-positive (including dopamine) cells. The following day, slices were washed five time in 1X PBS before a 2-hour secondary incubation, in 1:1000 Alexa Fluor 594-goat anti-mouse (*Biolegend*, Cat #405326) in PBS-T. Slices were then washed five times in 1X PBS before being mounted and allowed to dry. Mounting medium with DAPI (Vectashield, *Vector Laboratories*) was added to stain cell bodies, before adding the coverslip and sealing with nail polish. Slices were then imaged at 10x magnification using a confocal microscope (*Leica* DMi8) and LAS X software (*Leica*).

#### Q-learning model

Our Q-learning model was implemented using Matlab 2018a (*Mathworks*). The model considered a corridor of 30 discrete states where an agent would move sequentially through the states in each trial (*t*), and could choose to ‘lick’ or ‘not lick’ in each state (*s*). Cue states were defined as C∈[4, 9, 28] and the reward state $s_{R}=14$. If the agent chose to lick in the reward state, it would receives a reward with value =1. A threshold of licks allowed prior to the reward state was set to 2. If the agent exceeded this threshold prior to reaching the reward state, it received a reward value (*r*) of −0.1 and the trial was terminated such that it progressed to the first state of the next trial.

For each ‘true’ state $\left(s_{T}\right)$ visited, the agent estimated the state as: ${\hat{s}}_{T}=s_{T}+N\left(0\text{, }\;\sigma_{f}^{2}\right)$ using the standard normal distribution to inject a noise term using constant variance $\sigma_{f}^{2}$ set to 0.3, in order to simulate noisy visual observation (Lak et al., 2017, 2020). This estimate was used to construct a subjective normally-distributed belief ${\vec{\varphi }}_{s}$ of the agent’s position in the environment, weighted by uncertainty *u* that decreased in a Gaussian around the cue states, and a linear scale $\vec{\zeta }$ that simulated learning about the environment structure across the first 400 trials, such that each element of ${\vec{\varphi }}_{s}$ was calculated as: $\varphi_{s}\left(s\right)=\zeta \left(t\right)\cdot \left[e^{-\frac{{\left(s-{\hat{s}}_{T}\right)}^{2}}{2\sigma_{f}^{2}}}\cdot \left[1-u\left(s\right)\right]\right]$ where $\vec{u}$ confers the uncertainty relative to cue states. This is defined as $u\left(s\right)=\eta -\sum_{i}\eta \chi_{i}\left(s\right)$ where $\chi_{i}\left(s\right)=e^{-\frac{{\left(s-C_{i}\right)}^{2}}{2\sigma_{g}^{2}}}$ if ${\left|s-C\right|}_{i}<4$ and $\chi_{i}\left(s\right)=0$ otherwise. $C_{i}$ is a cue state where *i* is the index of cue states, and $\vec{\chi }$ is the sum of the Gaussian distributions around each cue state with amplitude η and standard deviation $\sigma_{g}$ (see Table S3). The belief state $s_{B}$ was then defined as the state where the belief distribution was maximal: $s_{B}=arg\;max_{s}\left({\vec{\varphi }}_{s}\right)$. Intuitively, this means that the belief is weighted by the relative certainty of the inference for that particular state due to presence or absence of nearby cues to inform/confirm its exact position.

Following determination of the belief state, *Q*-values for licking $\left(Q_{L}\right)$ and not licking $\left(Q_{N}\right)$ were weighted by the learning rate α, discount factor γ, and either the belief that the agent was in the reward state $\varphi_{s}\left(s_{R}\right)$ or the eligibility trace $\psi \left(s_{B}\right)$ respectively such that $Q_{L}\left(s_{B}\text{, }t\right)=\alpha \gamma \;Q_{L}\left(s_{B}\text{, }t-1\right)\;\varphi_{s}\left(s_{R}\right)$ and $Q_{N}\left(s_{B}\text{, }t\right)=\alpha \gamma \;Q_{N}\left(s_{B}\text{, }t-1\right)\;\psi \left(s_{B}\right)$. The reason we choose this weighting is because intuitively, the value of licking should be higher when you believe you are in the reward state, but conversely the value of not licking should be higher when you have just passed a cue, because that means you are not in the reward state. The eligibility trace was initialised at 0.5 and updated according to $\psi \left(s\right)=\text{min}\left\{1\text{, }\gamma \lambda \psi \left(s\right)+\varphi_{s}\left(s_{B}\right)\right\}\;\text{if}\;s\in C$ or ψ(s)=γλψ(s)ifs∉C or $\psi \left(s\right)=0\;\text{if}\;s=s_{R}$, where λ is the trace decay parameter.

Action (*a*) selection was performed in an ε-greedy manner where ε = 0.1, such that there was a 1−ε (90%) chance that action selection was performed by comparing the *Q*-values of licking and not licking and selecting whichever had a higher value: either $a=L\;\text{if}\;Q_{L}\left(s_{B}\text{, }t\right)>Q_{N}\left(s_{B}\text{, }t\right)$ or $a=N\;\text{if}\;Q_{L}\left(s_{B}\text{, }t\right)<Q_{N}\left(s_{B}\text{, }t\right)$. For the remaining ε (10%) probability, or if $Q_{L}$ and $Q_{N}$ were equal, actions were chosen randomly. The agent then received the outcome of the chosen action in that state. The expected maximal reward in the subsequent state, $Q_{max}$, was calculated using the cached values from the nearest preceding trial that had reached that state: $Q_{max}\left(s_{B}+1\text{, }t\right)=\text{max}\left[Q_{L}\left(s_{B}+1\text{, }t-1\right)\text{, }Q_{N}\left(s_{B}+1\text{, }t-1\right)\right]$. The TD error could then be calculated as $\delta \left(s_{B}\text{, }t\right)=r\left(s\right)+\gamma \;Q_{max}\left(s_{B}+1\text{, }t\right)-Q_{a}\left(s_{B}\text{, }t\right)$ and used to update the *Q*-value for the chosen action $\left(Q_{a}\right)$: $Q{\;}_{a}^{\text{′}}\left(s_{B}\text{, }t\right)\leftarrow Q_{a}\left(s_{B}\text{, }t\right)+\alpha \delta \left(s_{B}\text{, }t\right)$. The model then repeats this cycle for the next state, unless it exceeded the threshold of licks allowed prior to the reward state, in which case it moves to state 1 of the next trial.

We trained 100 agents for 4000 trials, where α = 0.9, γ = 0.95, λ = 0.92, 0≤ζ≤1. *Q*-values were initialised at 0.1. ‘Early’ trials were defined as the first 30, ‘mid’ trials were the next 70, and the rest were ‘late’. See Table S3 for full list of parameters and variables.

### Quantification and statistical analysis

Data presented include trials, sessions, and animals that met the criteria for analysis, detailed in the *Data preprocessing* section above. All fluorescence was z-scored across each session prior to analysis.

Continuous variables are shown as means with SEM. Boxplots indicate median in white, 25th and 75th percentiles as edges, and whiskers indicate most extreme points not considered outliers (outliers not shown). All statistical analyses are detailed in Table S1. Significance level achieved as indicated in figures is ^∗∗∗^: p < 0.001, ^∗∗^: p <0.01, ^∗^: p < 0.05 except where Bonferroni corrections are applied for comparisons across training stages, in which case ^∗∗∗^: p < 0.0003, ^∗∗^: p < 0.0033, ^∗^: p < 0.0167. Much of the data was not normally distributed (as indicated by cursory Shapiro-Wilk tests, not reported) so non-parametric tests were used. Where session averages are tested for significant difference from zero, unpaired Wilcoxon signed-rank tests were used. Where session averages of active and passive trial types were being compared, paired Wilcoxon signed-rank tests were used. Where session averages for each training stage were being compared, Mann-Whitney U tests were used and Bonferroni corrections applied.

Mean percentage trial type (Figure 1E) is an average over each training stage’s sessions for each of the eight animals. A Mann-Whitney U test was used to examine the difference between active and passive trial percentages for each learning stage, showing significant difference in late-stage training (p = 0.0078, n = 8). Comparison of pre-reward and reward zone mean licks/cm (Figure 1F) shows each mean values for each session plotted as individual points, with lines indicating best fit for all sessions in that training stage.

Data shown in Figures 2 and 3 show averages over sessions regardless of animal. Wilcoxon signed-rank tests (unpaired) were used to test whether data was significantly different from zero. Wilcoxon signed-rank tests (paired) are used to compare active and passive conditions, and Mann-Whitney U tests are used to compare different training stage data, followed by Bonferroni corrections for multiple comparisons. Linear mixed models are detailed in the text and figure legend (see STAR methods section and Table S2).

Model performance as shown by percentages of rewarded and unrewarded trials (Figure 4F) is averaged over all trials for each learning stage for 100 agents. Mann-Whitney U tests are used to compare trial type percentages for each training stage. Comparisons of lick distributions for late-stage trials following positive or negative slope trials (pooled across late-stage sessions and animals) (Figures 5C, 5D, 5F, and 5G) were performed using Mann-Whitney U tests for each spatial bin of 2 cm.

In Figure S2B, speed profiles are shown as averages over sessions regardless of animal. Wilcoxon signed-rank tests (paired) were performed on each 2 cm spatial bin to show significant differences between active and passive trials. In Figure 2C, each point shows average data for each session regardless of animal, with a line of best fit for each of the six combinations of training stage and trial type. Linear regression models (Matlab fitlm) were used to compare mean ramp gradient and mean rate of speed change for each condition.

Data in Figures S3 and S4 are shown over sessions regardless of animal. Wilcoxon signed-rank tests (unpaired) were used to test whether data was significantly different from zero. Wilcoxon signed-rank tests (paired) are used to compare active and passive conditions, and Mann-Whitney U tests are used to compare different training stage data with Bonferroni corrections. Linear mixed models are detailed in the figure legend.

Comparisons of lick distributions for late-stage trials following large positive, small positive, or negative reward response trials (pooled across late-stage sessions and animals) (Figure S8) were performed using Mann-Whitney U tests for each spatial bin of 2 cm.

#### Linear mixed models

Linear mixed modelling (LMM) with random intercepts and slopes was additionally implemented in Matlab (fitlme) to examine the dependence of the data on task variables such as trial type and session and account for repeated measures such that there were independent random effects terms for intercept and slope with animal identity as the grouping. Figures 2F, 2I, 2L, 3, S3F, S3G, S3J, and S4 used Models 1–3 to examine the effect of trial type and learning across sessions, while Figures 2C, S3C and S3E used Models 4–7 to examine the effects of trial type (being active vs passive or omission vs unrewarded) and condition (being pre- vs post-lick gradient or rewarded vs unrewarded). Models are denoted in Wilkinson notation where *y* is the variable of interest:

Model1:y∼session+(1|animal)+(−1+session|animal)+(−1+trialtype|animal)

Model2:y∼trialtype+session+(1|animal)+(−1+session|animal)+(−1+trialtype|animal)

Model3:y∼trialtype∗session+(1|animal)+(−1+session|animal)+(−1+trialtype|animal)

Model4:y∼condition+(1|animal)+(−1+session|animal)+(−1+trialtype|animal)+(−1+condition|animal)

Model5:y∼trialtype+(1|animal)+(−1+session|animal)+(−1+trialtype|animal)+(−1+condition|animal)

Model6:y∼condition+trialtype+(1|animal)+(−1+session|animal)+(−1+trialtype|animal)+(−1+condition|animal)

Model7:y∼condition∗trialtype+(1|animal)+(−1+session|animal)+(−1+trialtype|animal)+(−1+condition|animal)

Models were compared using a likelihood ratio test (Matlab compare) to ascertain which model best described the data. Table S2 indicates the results of these models.

## Data Availability

All data reported in this paper will be shared by the lead contact upon request. All original code has been deposited on GitHub (see key resources table) and is publicly available as of the date of publication. DOIs are listed in the key resources table. Any additional information required to reanalyse the data reported in this paper is available from the lead contact upon request. All procedures were conducted in accordance with the UK Animals Scientific Procedures Act (1986). Experiments were performed at University College London under personal and project licenses released by the Home Office following appropriate ethics review.

## Supplemental information

Document S1. Figures S1–S8 and Tables S1–S3

Document S2. Article plus supplemental information
