# Supplementary material for: Midbrain dopamine neurons signal phasic and ramping reward prediction error during goal-directed navigation
Source: Cell Rep. 2022 Oct 11;41(2):111470. doi: 10.1016/j.celrep.2022.111470 (PMC9631116; doi:10.1016/j.celrep.2022.111470)
Supplement: Document S1. Figures S1–S8 and Tables S1–S3 [file mmc1.pdf]

Cell Reports, Volume *41*

Supplemental information

Midbrain dopamine neurons signal phasic and ramping reward prediction error during goal-directed navigation

Karolina Farrell, Armin Lak, and Aman B. Saleem

1. Supplemental Information

1.1. Supplementary Figures

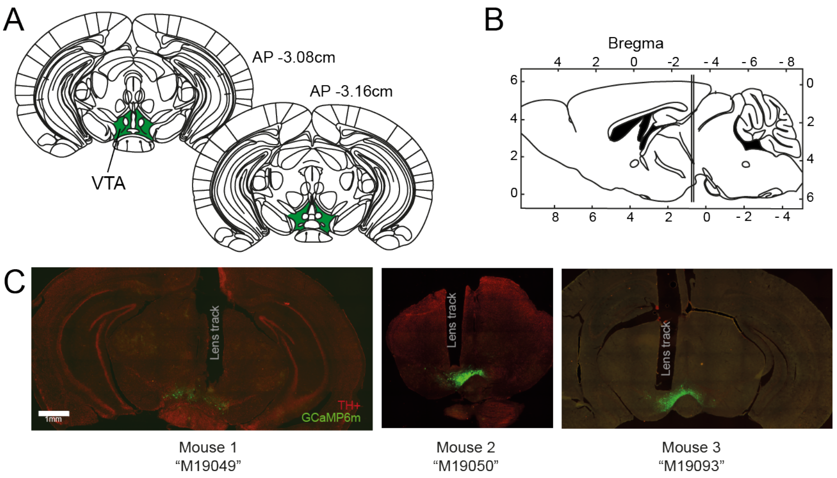

Figure S1: **Histology from example mice, Related to STAR Methods.** A) Figures 56 and 57 from Paxinos and Franklin (2001), showing diagram of horizontal section of mouse brain at -3.08cm at -3.16cm from bregma, with VTA highlighted in green. B) Inset of Figures 56 and 57 from Paxinos and Franklin (2001) showing diagram of sagittal section of mouse brain, with sections at -3.08cm and -3.16cm from bregma indicated. C) Example histology from three mice, showing GCaMP6m (green) and tyrosine hydroxylase (TH) staining (red).

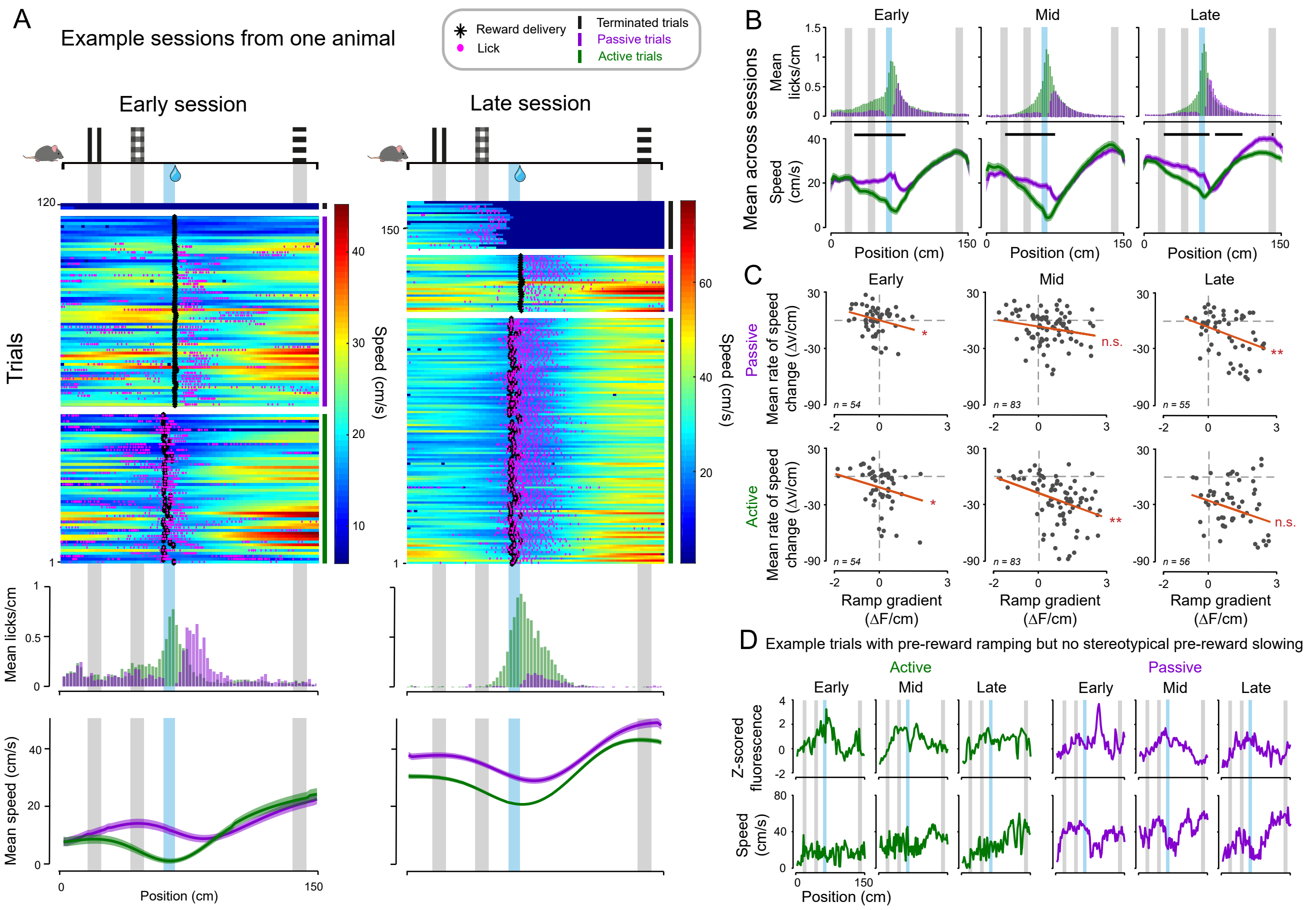

Figure S2: **Behaviour and speed analysis, Related to Figure 1.** A) Example early and late sessions for one animal sorted for trial type, showing speed, reward delivery (black asterisks) and licks (circles). Below are mean licks per cm and mean speed for each session for active (green) and passive (purple) trials. B) Mean lick distribution and speed profile across sessions per training stage for active and passive trials. Black bars indicate significant difference between active and passive speed profiles for positions indicated (early-stage: n=55, $p<0.001$ for bins 30, 34, 38-78cm, $p<0.01$ for bins 26-28, 32, 36, 80cm, $p<0.05$ for bin 82cm, mid-stage: n=83, $p<0.001$ for bins 28-74cm, $p<0.01$ for bin 26cm, $p<0.05$ for bins 10, 22, 24, 76cm, late-stage: n=55, $p<0.001$ for bins 34-70, 80, 84, 90-92cm, $p<0.01$ for bins 24-32, 78, 82, 86-88, 94-98, 104cm, $p<0.05$ for bins 22, 72, 100-102, 108, 140-142cm, Wilcoxon signed rank test). C) Gradient of fitted line to change in speed over pre-reward distance (mean rate of pre-reward speed change, calculated as change in speed per cm) plotted against gradient of fitted line to pre-reward calcium activity per session for each training stage for passive (top) and active (bottom) trials. Line fitted to points using Matlab polyfit shown in red. Linear regression models were performed for each plot: passive early (R^2=0.0812, p=0.0367, n=54), mid (R^2=0.0391, p=0.0731, n=83), late (R^2=0.1332, p=0.0062, n=56), active early (R^2=0.1069, p=0.0158, n=54), mid (R^2=0.1088, p=0.0023, n=83), and late (R^2=0.0367, p=0.1572, n=55). D) Example trials for each trial type and in each training stage where a pre-reward ramp exists without the stereotypical pre-reward slowing, indicating that pre-reward slowing is not a prerequisite for pre-reward ramping.

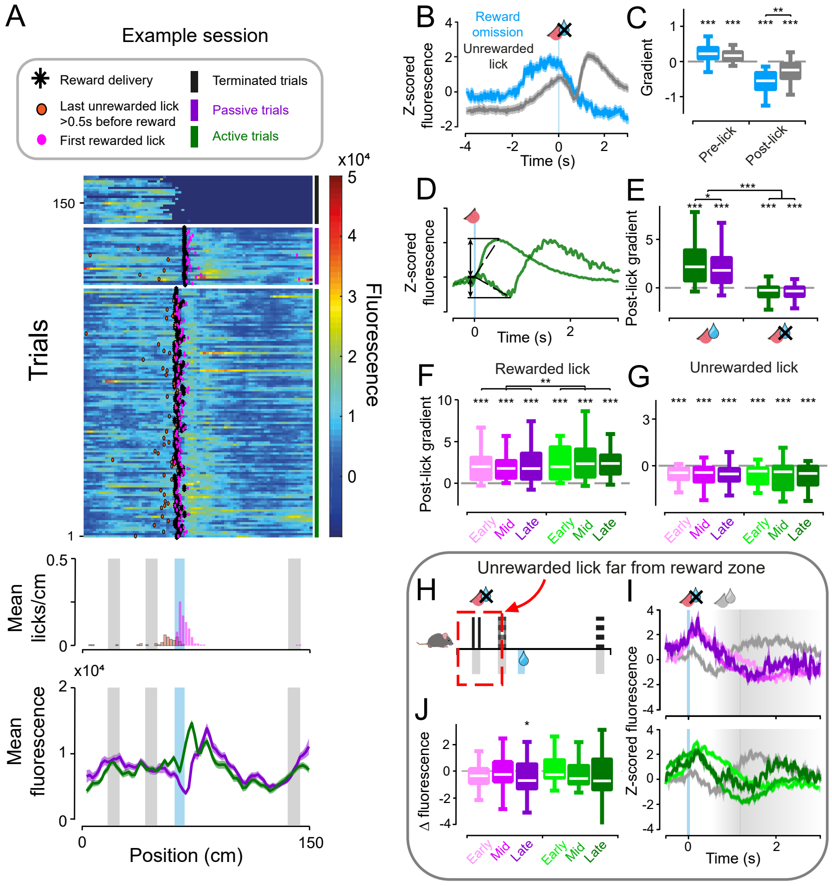

Figure S3: **Additional characterisation of post-lick responses, related to Figure 2.** A) Example session showing fluorescence across trials (sorted by trial type) and locations of last unrewarded lick occurring at least 0.5s before reward delivery (orange dots) and first rewarded lick occurring after reward delivery (pink dots). Lick distribution and fluorescence are averaged below. B) Mean activity traces averaged over sessions from reward omission trials aligned to time of first lick in reward zone (blue) and unrewarded licks with only one lick occurring before the reward zone (and reward following later in the trial) from the same sessions as the omission trials (grey). C) Comparison of pre-lick gradient (calculated by fitting a line to activity in the window of -3s to time of aligned lick) and post-lick gradient for the traces shown in B. Pre-lick gradients are significantly greater than zero, whereas post-lick response gradients are significantly lower than zero ($p<0.0001$ for all, n=37 (omission, pre-lick), n=50 (unrewarded, pre-lick), n=35 (omission, post-lick), n=39 (unrewarded, post-lick), Wilcoxon signed rank). The post-lick gradients are also significantly different between the reward omission trials and the unrewarded trials ($p=0.0427$, n=35, Wilcoxon signed rank), indicative of greater expectation at the time of lick (in the reward zone) in the omission trace compared to unrewarded lick (before the reward zone). LMM analysis confirmed a significant effect of pre- vs post-lick condition (Model4: $p<0.001$, b=0.6572 95% CI [0.3004,1.014], t=3.6359) but not of omission vs unrewarded trial type. D) Schematic of how change in fluorescence and lines are fitted to mean of activity traces from active trials following rewarded and unrewarded licks. E) Boxplots of gradient of slope following lick for both rewarded and unrewarded licks, as shown in Figure 2A-B. Asterisks indicate significant difference from zero ($p<0.0001$ for all, n=188 (passive, rewarded), n=176 (active, rewarded), n=187 (passive, unrewarded), n=193 (active, unrewarded), Wilcoxon signed rank). Difference between gradient of slope for rewarded licks in active and passive trials is significant ($p=0.0376$, n=176, Mann-Whitney U test), as is the gradient of the slope following rewarded licks vs unrewarded licks ($p<0.0001$, n=191, Mann-Whitney U test). LMM analysis confirmed that trial type and rewarded vs unrewarded condition are significant (Model6: $p=0.0092$, b=-0.28996 95% CI [-0.5079,-0.071998], t=-2.6117 and $p<0.0001$, b=-2.8167 95% CI [-3.4045,-2.229], t=-9.4089 respectively) but not the interaction between them. F-G) Boxplots of gradient of post-lick response following rewarded and unrewarded licks, split by training stage, corresponding to Figure 2D-E. All are significantly different from zero ($p<0.0001$ for all, n=54, 82, 52 (passive, rewarded, early-mid-late respectively), n=45, 78, 53 (active, rewarded, early-mid-late respectively), n=48, 57, 36 (passive, unrewarded, early-mid-late respectively), n=31, 43, 33 (active, unrewarded, early-mid-late respectively), Wilcoxon signed rank). Mean gradient of post-rewarded lick response is significantly greater in active compared to passive ($p=0.0075$, n=176, Wilcoxon signed rank). LMM analysis confirmed that only the trial type is significant for post-rewarded lick gradients (Model5: $p=0.03015$, b=-0.47859 95% CI [-0.91098,-0.046196], t=-2.1766) but neither trial type nor session is significant for post-unrewarded lick gradients. H) Schematic showing that unrewarded licks far from the reward zone as indicated in I) were before 45cm into the corridor, where the first lick from each trial is used. I) VTA dopaminergic activity as a function of time following early unrewarded licks for passive (purple) and active (green) trials, with SEM shown by semi-transparent areas. J) Boxplots of change in fluorescence following early unrewarded licks (maximum difference in window of 0-0.6s following lick). Asterisks indicate significant difference from zero ($p<0.05$ for late passive, n=32, Wilcoxon signed rank). LMM analysis confirmed a significant effect of session (Model1: $p=0.011921$, b=-0.026452 95% CI [-0.047023,-0.005881], t=-2.5317).

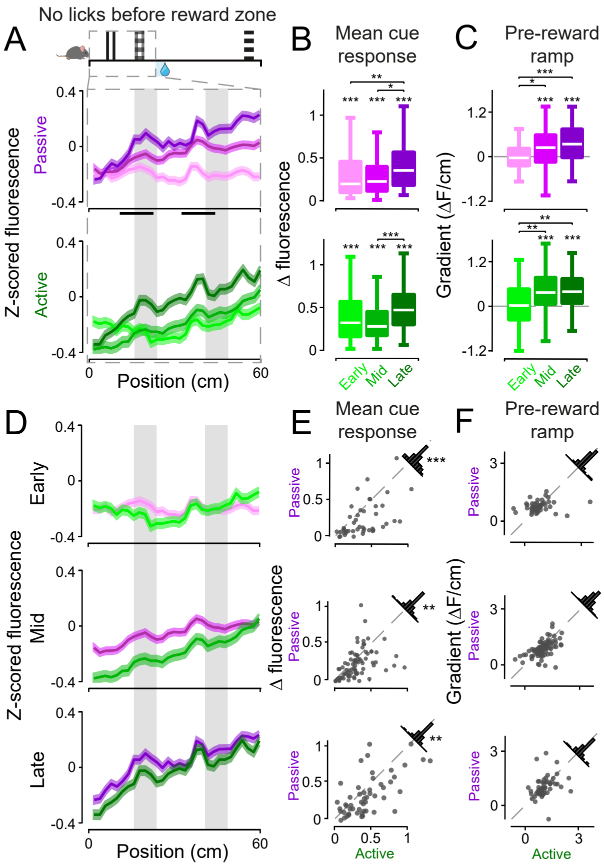

Figure S4: **Pre-reward activity in trials that had no licking prior to the reward zone, Related to Figure 3.** A) Mean activity traces from trials where no licking occurred prior to the reward zone, focusing on the pre-reward corridor region from 0-60cm, split into different training stages. Black bars indicate position windows where cue responses are calculated for use in B. B) As in Figure 3B, boxplots of the mean of maximal change in fluorescence for the two cue windows indicated by the black bars in A, split by training stage. Values from each trial are averaged over each session. All distributions are significantly larger than zero ($p<0.0001$ for all, n=54, 83, 54 (passive, early-mid-late respectively), n=45, 78, 54 (active, early-mid-late respectively), Wilcoxon signed rank test). Change in fluorescence for passive trials is significantly different between early- and late-stage training, as well as mid- and late-stage training ($p=0.003$ and $p=0.005$ respectively, n=54, Mann-Whitney U test, Bonferroni corrections applied). For active trials, change in fluorescence is significantly different between mid- and late-stage training ($p=7.6575e-05$, n=54, Mann-Whitney U test, Bonferroni corrections applied). LMM analysis confirmed that trial type and session both have significant effects on mean cue response (Model2: $p=0.00268$, $b=-0.06967$ 95% CI [-0.115,-0.0243], $t=-3.0224$ and $p=0.000275$, $b=0.00569$ 95% CI [0.00265,0.00874], $t=3.6735$ respectively). C) Boxplots of the mean pre-reward ramp gradient, calculated by fitting a line to activity in the 0-60cm window. Asterisks above mid- and late-stage boxplots for active and passive trials indicate distribution is significantly greater than zero ($p<0.001$ for all, n=83 (passive, mid), n=54 (passive, late), n=78 (active, mid), n=54 (active, late), Wilcoxon signed rank test). Pre-reward ramp gradient for passive trials is significantly different between early- and mid-stage training, and early- and late-stage training ($p=0.0049$ and $p=2.1912e-04$ respectively, n=54, Mann-Whitney U test, Bonferroni corrections applied). For active trials, ramp gradient is significantly different between early- and mid-stage training as well as early- and late-stage training ($p=0.0013$ and $p=0.0014$ respectively, n=45, Mann-Whitney U test). LMM analysis confirmed that trial type and session both have significant effects on ramp gradient (Model2: $p=0.0277$, $b=-0.00203$ 95% CI [-0.00384,-0.000224], $t=-2.2099$ and $p=6.1766e-05$, $b=0.0003364$ 95% CI [0.000215,0.000458], $t=5.4463$ respectively). D-F) Same data shown in A-C, but directly comparing passive and active for each training stage. For E, significant differences are found between active and passive mean change in fluorescence per session for the two cues at early-, mid- and late-stage training ($p<0.001$ and n=45, $p=0.0042$ and n=58, $p=0.0026$ and n=73 respectively, Wilcoxon signed rank test).

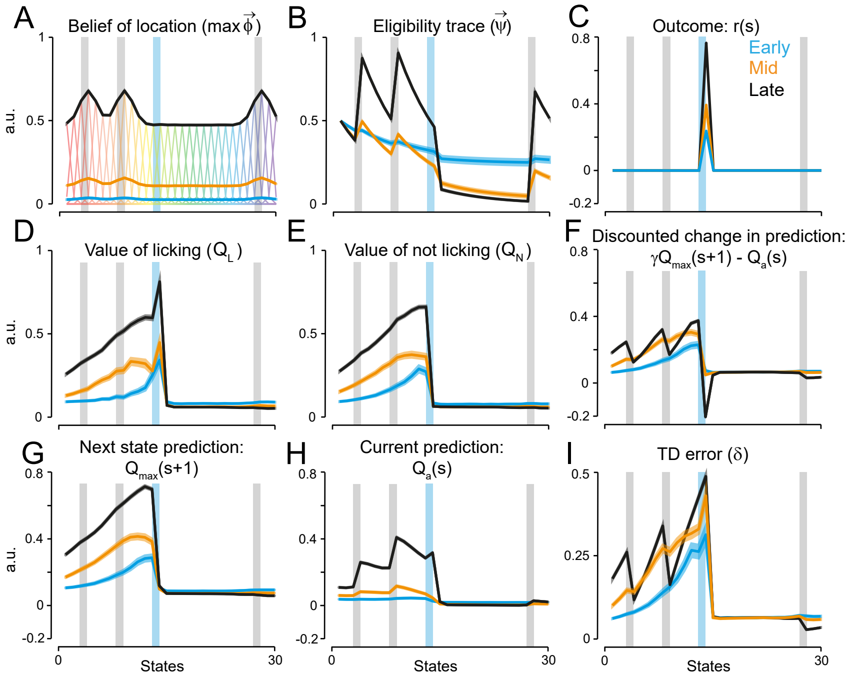

Figure S5: **Additional model outputs, Related to Figure 4.** A) Subjective belief distributions $\vec{\phi}_s$ are summarised as their maximum values for each state. The mean of early trials is summarised in blue, mid in orange, and late in black. For the late trials, mean belief distributions across trials and agents are shown for each state in rainbow colours, in reference to the state colours in Figure 4. Cue states are shown in grey and the reward state in light blue. B) Mean eligibility trace ψ is shown for each learning stage. C) Mean outcome (reward value) per training stage. D-E) Q-values for licking and not licking respectively, averaged over each learning stage. F-H) Mean discounted change in prediction, next state maximal prediction of value, and current prediction of value based on the chosen action respectively, split for each learning stage. I) TD error (δ) across learning stage, as in Figure 4, shown here for comparison with C) and F). J) Trials from one example agent showing TD error (δ), split into learning stages and sorted by trial type. Reward delivery is indicated with black asterisks and licks with green dots. Mean across trials from licks/state and TD error are indicated below.

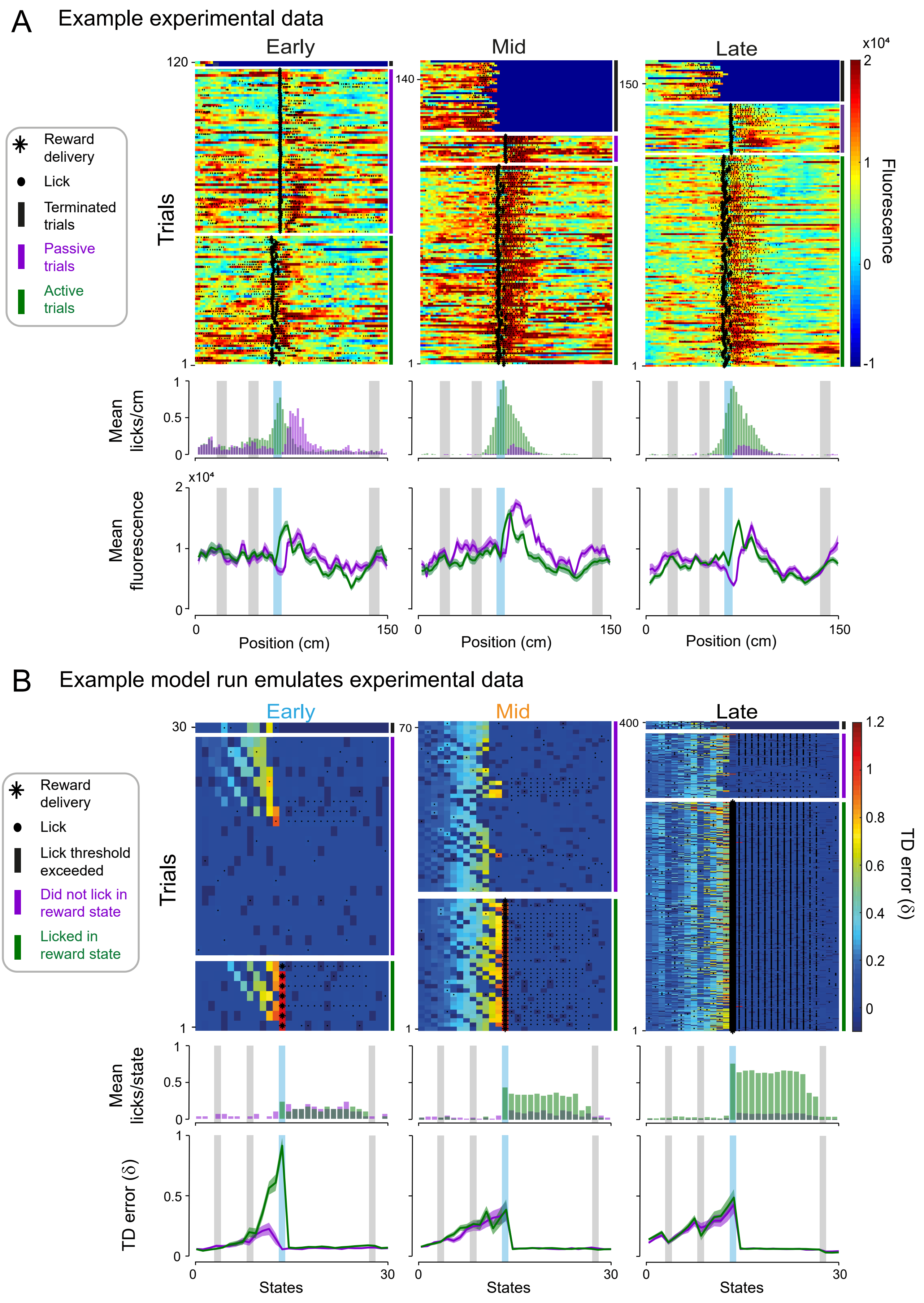

Figure S6: **Observed VTA dopamine neuron activity is similar to model's TD error across training, Related to Figure 4.** A) Example early, mid, and late sessions from one mouse, showing observed fluorescence across trials (sorted by trial type), reward delivery (black asterisks), and licks (black dots), with mean lick distributions and fluorescence traces for each session indicated below, split for active (green) and passive (purple) trials. B) Example model run from one agent showing TD error across trials (sorted by trial type), reward delivery (black asterisks), and licks (pink dots), with mean licks per state and TD error traces for each learning stage indicated below, split for trials where the agent licked in the reward state (green) and did not lick in the reward state (purple).

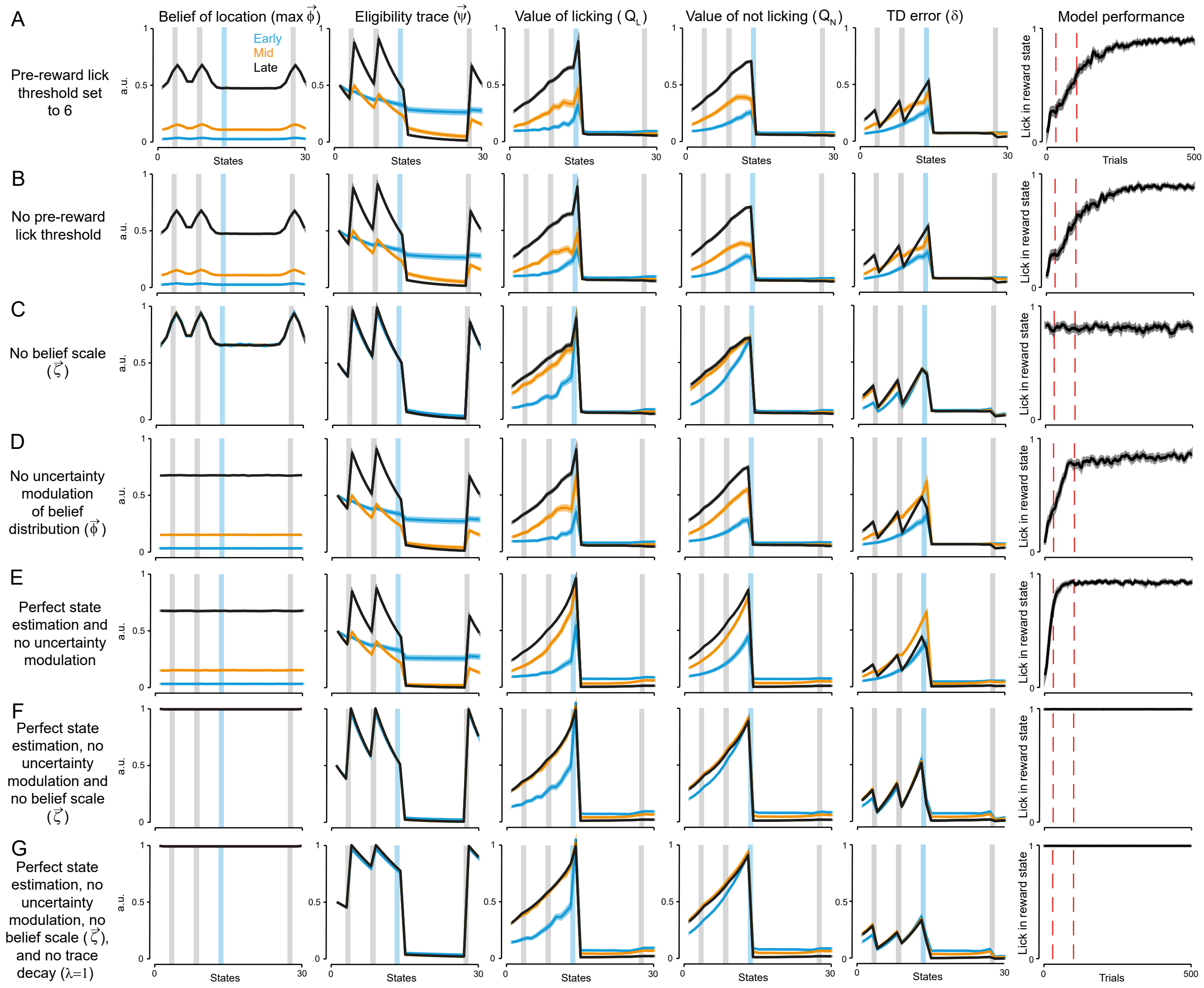

Figure S7: **Results of altering model, Related to Figure 4.** A) Model outputs when pre-reward lick threshold was set to 6 licks across learning for 100 agents, compared to 2 licks as shown in Figures 4 and 5. Model outputs include belief of location (maximum of belief distribution per state visited, $max(\vec{\phi})$), eligibility trace ($\vec{\psi}$), value of licking (Q_L) and not licking (Q_N), TD error (δ), and model performance over the first 500 trials (as determined by licking in the reward state or not). Mean of early trials is shown in blue, mid trials in orange, and late trials in black. Cue states are shown in grey and the reward state in light blue. Red dashed lines indicate the separations between different learning stages. B) Model outputs when no pre-reward lick threshold is imposed. C) Model outputs when no belief scaling ($\vec{\zeta}$) is imposed. D) Model outputs when no weighting of the belief distribution ($\vec{\phi}_s$) by uncertainty ($\vec{u}$) is imposed. E) Same as D) but also with perfect state estimation assumed (belief state s_B is set to the true current state s_T). F) Same as E) but also with no belief scaling ($\vec{\zeta}$) imposed. G) Same as F) but also with the trace decay parameter λ set to 1.

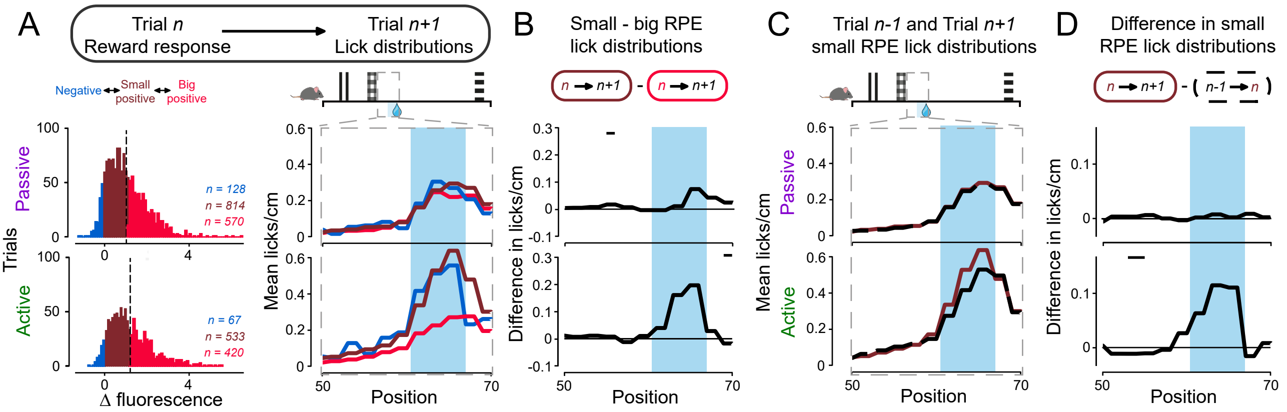

Figure S8: **Reward response improves task performance on subsequent trial, Related to Figure 5.** A) Left: Distributions of post-reward delivery reward responses (RPEs) per late-stage training trial n with no licks prior to reward zone, for passive (top) and active (bottom). Groups are big positive RPE trials (red), small positive RPE trials (brown) and negative RPE trials (blue). Right: Distributions of pre-reward licks on trials following big positive RPE trials (red), small positive RPE trials (brown) and negative RPE trials (blue), focusing on 50-70cm in the virtual corridor. B) Difference between lick distributions shown in A) right for passive (top) and active (bottom) trials. Black bars denote a significant difference between distributions (for passive p=0.0253, n=570 for 55-56cm, for active p=0.0229, n=420 for 69-70cm, Mann-Whitney U test). C) Distributions of pre-reward licks on trials following small positive RPE trials (brown) and preceding small positive RPE trials (black, dashed). D) Difference between lick distributions shown in C for passive (top) and active (bottom) trials (for active p=0.0048, n=531 for 63-64cm, Mann-Whitney U test).

1.2. Tables

Table S1: List of statistical tests shown in main figures

M-W: Mann-Whitney U test (paired);

W(u): Wilcoxon signed rank test (unpaired);

W(p): Wilcoxon signed rank test (paired)

For Figures 2 and 3, statistical tests are performed across sessions. For Figures 4 and 5, statistical tests are performed across trials.

Fig	Variables	p-value	n	Test
1E	Late-stage percentage passive trials, late-stage percentage active trials	0.0078	8 animals	M-W
2C	Active post-rewarded lick responses	8.8950e-24	176 sessions	W(u)
2C	Passive post-unrewarded lick responses	1.4454e-07	141 sessions	W(u)
2C	Active post-unrewarded lick responses	2.2765e-10	107 sessions	W(u)
2C	Passive post-rewarded lick responses, active post-rewarded lick responses	0.0155	194 sessions	W(p)
2C	Post-rewarded lick responses, post-unrewarded lick responses	2.7494e-22	194 sessions	W(p)
2F	Early-stage passive post-rewarded lick responses	6.3378e-09	54 sessions	W(u)
2F	Mid-stage passive post-rewarded lick responses	7.2065e-12	82 sessions	W(u)
2F	Late-stage passive post-rewarded lick responses	1.1845e-05	52 sessions	W(u)
2F	Early-stage active post-rewarded lick responses	6.2459e-07	45 sessions	W(u)
2F	Mid-stage active post-rewarded lick responses	1.5766e-10	78 sessions	W(u)
2F	Late-stage active post-rewarded lick responses	3.8267e-09	53 sessions	W(u)
2F	Late-stage passive post-rewarded lick responses, late-stage active post-rewarded lick responses	0.0406	52 sessions	W(p)
2I	Early-stage passive post-unrewarded lick responses	2.8253e-04	48 sessions	W(u)
2I	Mid-stage passive post-unrewarded lick responses	0.0012	57 sessions	W(u)
2I	Late-stage passive post-unrewarded lick responses	6.5155e-04	36 sessions	W(u)
2I	Late-stage active post-unrewarded lick responses	0.0044	33 sessions	W(u)
2I	Early-stage active post-unrewarded lick responses, late-stage active post-unrewarded lick responses	0.0078	31, 33 sessions	M-W
2I	Mid-stage active post-unrewarded lick responses, late-stage active post-unrewarded lick responses	0.0352	43, 33 sessions	M-W
2L	Early-stage passive post-reward responses	2.2765e-10	54 sessions	W(u)
2L	Mid-stage passive post-reward responses	2.7928e-15	83 sessions	W(u)
2L	Late-stage passive post-reward responses	2.9637e-10	55 sessions	W(u)
2L	Early-stage active post-reward responses	1.9244e-10	54 sessions	W(u)
2L	Mid-stage active post-reward responses	3.3498e-15	83 sessions	W(u)

2L	Late-stage active post-reward responses	7.7267e-10	56 sessions	W(u)
2L	Mid-stage passive post-reward responses, late-stage passive post-reward responses	0.0237	83, 55 sessions	M-W
2L	Early-stage active post-reward responses, late-stage active post-reward responses	4.0522e-04	54, 56 sessions	M-W
2L	Mid-stage active post-reward responses, late-stage active post-reward responses	0.0039	83, 56 sessions	M-W
3B	Early-stage passive mean cue responses	1.6257e-10	54 sessions	W(u)
3B	Mid-stage passive mean cue responses	2.5034e-15	83 sessions	W(u)
3B	Late-stage passive mean cue responses	1.1076e-10	55 sessions	W(u)
3B	Early-stage passive mean cue responses, late-stage passive mean cue responses	1.0094e-05	54, 55 sessions	M-W
3B	Mid-stage passive mean cue responses, late-stage passive mean cue responses	1.0300e-04	83, 55 sessions	M-W
3B	Early-stage active mean cue responses	1.6257e-10	54 sessions	W(u)
3B	Mid-stage active mean cue responses	2.5034e-15	83 sessions	W(u)
3B	Late-stage active mean cue responses	7.5475e-11	56 sessions	W(u)
3B	Early-stage active mean cue responses, mid-stage active mean cue responses	0.0169	54, 83 sessions	M-W
3B	Early-stage active mean cue responses, late-stage active mean cue responses	5.3701e-05	54, 56 sessions	M-W
3B	Mid-stage active mean cue responses, late-stage active mean cue responses	0.0015	83, 56 sessions	M-W
3C	Early-stage passive ramp gradients	0.0185	54 sessions	W(u)
3C	Mid-stage passive ramp gradients	1.2954e-04	83 sessions	W(u)
3C	Late-stage passive ramp gradients	3.9987e-09	55 sessions	W(u)
3C	Early-stage passive ramp gradients, mid-stage passive ramp gradients	1.4195e-05	54, 83 sessions	M-W
3C	Early-stage passive ramp gradients, late-stage passive ramp gradients	6.0579e-09	54, 55 sessions	M-W
3C	Mid-stage passive ramp gradients, late-stage passive ramp gradients	0.0212	83, 55 sessions	M-W
3C	Mid-stage active ramp gradients	2.9572e-09	83 sessions	W(u)
3C	Late-stage active ramp gradients	5.4663e-09	56 sessions	W(u)
3C	Early-stage active ramp gradients, mid-stage active ramp gradients	8.6079e-08	54, 83 sessions	M-W
3C	Early-stage active ramp gradients, late-stage active ramp gradients	2.7530e-08	54, 56 sessions	M-W
3F	Early-stage active ramp gradients, early-stage passive ramp gradients	0.0243	54 sessions	W(p)
3F	Mid-stage active ramp gradients, mid-stage passive ramp gradients	1.3368e-05	83 sessions	W(p)
3F	Late-stage active ramp gradients, late-stage passive ramp gradients	0.0426	55 sessions	W(p)
4C	Early-stage percentage unrewarded trials, early-stage percentage rewarded trials	6.3896e-16	100 agents	M-W
4C	Mid-stage percentage unrewarded trials, mid-stage percentage rewarded trials	1.7324e-05	100 agents	M-W
4C	Late-stage percentage unrewarded trials, late-stage percentage rewarded trials	3.8887e-18	100 agents	M-W

5C/D	Late-stage zero licks before reward passive positive slope subsequent trial lick distribution, late-stage zero licks before reward passive negative slope subsequent trial lick distribution: bin 61-62cm, bin 67-68cm	0.0116, 0.0009	568, 392 trials	M-W
5C/D	Late-stage zero licks before reward active positive slope subsequent trial lick distribution, late-stage zero licks before reward active negative slope subsequent trial lick distribution: bin 63-64cm, bin 65-66cm, bin 67-68cm	0.0283, 0.0032, 0.0403	398, 262 trials	M-W
5F/G	Late-stage zero licks before reward passive positive slope subsequent trial lick distribution, late-stage zero licks before reward passive positive slope previous trial lick distribution: bin 61-62cm	0.0236	568, 564 trials	M-W
5F/G	Late-stage zero licks before reward active positive slope subsequent trial lick distribution, late-stage zero licks before reward active positive slope previous trial lick distribution: bin 63-64cm	0.0174	398, 398 trials	M-W

Table S2: List of linear mixed model results shown in main figures

Model refers to best model out of Models1-3 or Models4-7 from likelihood ratio tests. Factor indicates the data that is contrasted against the intercept, with corresponding beta coefficient estimate (indicating the fixed effect of the factor), 95% confidence intervals for the beta estimate, the t-statistic, and the random effect of the animal given by intercept standard deviation and its 95% confidence intervals.

**Fig**	**Model**	**Factor**	**p-value**	**beta**	**95% CI**	**tStat**	**Animal intercept std**	**95% CI**
2C	4	Unrewarded	2.6965e-25	-1.4713	-1.7371, -1.2056	-10.874	0.2005	0.05929, 0.67803
2I	1	Session	0.002021	-0.030456	-0.04968, -0.01123	-3.1205	1.8148e-10	0.00103, 0.08626
2L	3	Session	0.0378	-0.021644	-042061, -0.001226	-2.8084	0.33608	0.18117, 0.62347
		Passive	0.88565	-0.016584	-0.24318, 0.21001	-0.1439		
		Session: Passive	0.41013	0.0051042	-0.007067, 0.017275	0.8246		
3B +E	1	Session	0.00013887	0.0082594	0.004040, 0.012478	3.8491	0.11294	0.06629, 0.19244
3C +F	2	Passive	0.014617	-0.0020573	-0.003706, -0.0004082	-2.4529	0.0033033	0.001763, 0.006190
		Session	1.7061e-06	0.00047162	0.0002809, 0.0006624	4.8613		
S3C	4	Post-lick	0.00036569	0.6572	0.3004, 1.014	3.6359	0.25854	0.10108, 0.6613
S3E	6	Passive	0.0091925	-0.28996	-0.50791, -0.071998	-2.6117	0.66019	0.35688, 1.2213
		Unrewarded	6.1479e-20	-2.8167	-3.4045, -2.229	-9.4089		
S3F	5	Passive	0.030153	-0.47859	-0.91098, -0.046196	-2.1766	0.71827	0.66754, 0.77285
S3J	1	Session	0.011921	-0.026452	-0.04702, -0.005881	-2.5317	1.9861e-09	NaN
S4B +E	2	Passive	0.0039429	-0.072272	-0.12126, -0.023285	-2.9012	0.13928	0.07577, 0.25602
		Session	0.025129	0.0078105	0.0009801, 0.014641	2.2487		
S4C +F	2	Passive	0.015186	-0.0022945	-0.004144, -0.0004448	-2.4394	0.0050702	0.003250, 0.00791
		Session	0.00070721	0.00033723	0.0001431, 0.0005314	3.4159		

Table S3: Model variables

Variable	Dimensions	Description	Value
a	scalar	chosen action	
A	1 × number of actions	set of possible actions	
α	scalar	learning rate step-size parameter	0.9
C	1 × number of cue states	set of cue states	
δ	states × trials	temporal difference error	
ϵ	scalar	parameter determining greediness of action selection	0.1
$\vec{\zeta}$	1 × 400	linear scale for belief weighting for first 400 trials	
η	scalar	amplitude of Gaussian distribution for uncertainty around each cue state	0.3
γ	scalar	discount factor step-size parameter	0.95
i	scalar	index of cue states	
λ	scalar	trace decay parameter	0.92
Q_a	states × trials	Q-value of chosen action	
Q_L	states × trials	Q-value of licking	
Q_N	states × trials	Q-value of not licking	
Q_{max}	states × trials	maximum Q-value of either action	
r	1 × states	reward	1
s	scalar	state	
s_B	scalar	belief state	
s_R	scalar	reward state	
s_T	scalar	true state	
$\hat{s}_T$	scalar	estimated true state (observed state)	
σ_f^2	scalar	variance of normal distribution for s_T estimation and construction of belief	0.3
σ_g^2	scalar	variance of Gaussian distribution for uncertainty calculation	1.2
t	scalar	trial	
$\vec{u}$	1 × states	uncertainty across states	
$\vec{\phi}_s$	1 × states	for state s, the belief distribution of what state the agent is located in across all states	
Φ	states × states	matrix of belief distributions across states for each state visited	
$\vec{\psi}$	1 × states	eligibility trace-like representation of cues / indicator of upcoming reward	
$\vec{\chi}$	1 × states	sum of the Gaussian distributions around each cue state	
$\vec{z}$	1 × states	eligibility trace	
